# Group 3 Innate Lymphoid Cells Exacerbate Lupus Nephritis by Promoting B Cell Activation in Kidney Ectopic Lymphoid Structures

**DOI:** 10.1002/advs.202302804

**Published:** 2023-11-01

**Authors:** Feng Li, Zhou Liang, Haojie Zhong, Xinrong Hu, Ziwen Tang, Changjian Zhu, Jiani Shen, Xu Han, Ruoni Lin, Ruilin Zheng, Ruihan Tang, Huajing Peng, Xunhua Zheng, Chengqiang Mo, Peisong Chen, Xin Wang, Qiong Wen, Jianbo Li, Xi Xia, Hongjian Ye, Yagui Qiu, Jianwen Yu, Dongying Fu, Jiaqi Liu, Rong Wang, Huixin Xie, Yun Guo, Xiaoyan Li, Jinjin Fan, Qinghua Liu, Haiping Mao, Wei Chen, Yi Zhou

**Affiliations:** ^1^ Department of Nephrology The First Affiliated Hospital, Sun Yat‐sen University Guangzhou 510080 China; ^2^ NHC Key Laboratory of Clinical Nephrology (Sun Yat‐Sen University) and Guangdong Provincial Key Laboratory of Nephrology Guangzhou 510080 China; ^3^ Department of Hepatobiliary and Pancreatic Surgery The First Affiliated Hospital, Shenzhen University Shenzhen 518000 China; ^4^ Department of Urology The First Affiliated Hospital, Sun Yat‐sen University Guangzhou 510080 China; ^5^ Department of Laboratory Medicine The First Affiliated Hospital, Sun Yat‐sen University Guangzhou 510080 China

**Keywords:** B cells, ectopic lymphoid structures, group 3 innate lymphoid cells, lupus nephritis

## Abstract

Group 3 innate lymphoid cells (ILC3s) represent a new population in immune regulation, yet their role in lupus nephritis (LN) remains elusive. In the present work, systemic increases in ILC3s, particularly in the kidney, are observed to correlate strongly with disease severity in both human and murine LN. Using MRL/*lpr* lupus mice and a nephrotoxic serum‐induced LN model, this study demonstrates that ILC3s accumulated in the kidney migrate predominantly from the intestine. Furthermore, intestinal ILC3s accelerate LN progression, manifested by exacerbated autoimmunity and kidney injuries. In LN kidneys, ILC3s are located adjacent to B cells within ectopic lymphoid structures (ELS), directly activating B cell differentiation into plasma cells and antibody production in a Delta‐like1 (DLL1)/Notch‐dependent manner. Blocking DLL1 attenuates ILC3s’ effects and protects against LN. Altogether, these findings reveal a novel pathogenic role of ILC3s in B cell activation, renal ELS formation and autoimmune injuries during LN, shedding light on the therapeutic value of targeting ILC3s for LN.

## Graphical abstract

In lupus nephritis (LN), gut‐derived group 3 innate lymphoid cells (ILC3s) migrate from the small intestine to the kidney via CXCR6/CXCL16 axis, and contribute to the progression of LN, including spleen and lymph node enlargement, antibody production and renal injury. In renal ectopic lymphoid structures (ELS), ILC3s induce B cell activation in a Delta‐like1 (DLL1)/Notch‐dependent manner, accompanied by up‐regulation of Deltex1 and MAML1.

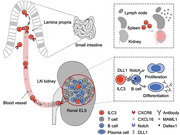

## 1 Introduction

Systemic lupus erythematosus (SLE) is a life‐threatening autoimmune disease characterized by aberrant activity of the immune system and dysfunction of various organs.^[^ ^1^ ^]^ Lupus nephritis (LN), one of the most severe complications of SLE, affects ≈50% of patients with SLE and results in high morbidity and mortality among these patients.^[^ ^1^, ^2^ ^]^ The etiology of LN involves B cell‐derived antibodies binding to intrarenal autoantigens, deposition of immune complexes and complement, and subsequent inflammatory responses in the kidney.^[^ ^3^ ^]^ As renal immunopathology progresses, infiltrating leukocytes aggregate and form ectopic lymphoid structures (ELS), which maximize the encounter between self‐antigens and immune effector cells, leading to a more robust B cell reaction.^[^ ^4^ ^]^ Despite the evolving understanding of the mechanisms underlying LN, the exact pathogenesis, including those triggering local B cell responses and ELS formation, has yet to be fully clarified and potential players may remain unrecognized, limiting the development of new therapeutic strategies for the disease.

Innate lymphoid cells (ILCs), a novel group of innate immune cells, have been found to play critical roles in diverse immune‐related diseases,^[^ ^5^ ^]^ such as inflammatory bowel disease,^[^ ^6^ ^]^ allergic asthma,^[^ ^7^ ^]^ and psoriasis.^[^ ^8^ ^]^ Since their activations do not require distinct signals from antigen‐presenting cells, ILCs rapidly respond to host or microbial stimuli and promote subsequent innate and adaptive immune responses.^[^ ^5a^ ^]^ Based on their transcription factor expression profile, ILCs are mainly divided into three subgroups, including T‐bet^+^ ILC1s, GATA3^+^ ILC2s, and RORγt^+^ ILC3s.^[^ ^5^ ^]^ Emerging data have shown marked alterations of ILC subsets in SLE, but detection is limited to peripheral blood samples from patients,^[^ ^9^ ^]^ and little is known about their respective roles in the disease or in specific organ involvement. Apart from an earlier study that found a possible protective role of ILC2s in LN,^[^ ^10^ ^]^ little progress has been made so far. The relevance between ILC3s and LN has not emerged until recently when a study focusing on pathogenic factors in LN mentioned ILC3s as a possible source.^[^ ^11^ ^]^ However, the exact role of ILC3s in LN remains unknown.

In this study, we uncovered that systemic ILC3s were elevated and positively associated with disease severity in human and murine LN. Among ILC3s increased in various tissues, kidney‐ and gut‐localized ILC3s showed an intrinsic link with the most similar transcriptomics during LN development. Intestinal CXCR6^+^ ILC3s were shown to potentially migrate to lupus kidneys as a major source of increased renal ILC3s and in turn accelerated LN progression. Mechanistically, ILC3s promoted B cell proliferation, differentiation and function through the Notch ligand Delta‐like1 (DLL1), and contributed to ELS formation in the kidney, eventually leading to aggravated systemic autoimmune responses and renal injury. These findings highlight the important role of ILC3s in LN and their potential as novel therapeutic targets.

## 2 Results

### 2.1 ILC3s are Increased in PBMC and Kidneys of LN Patients

**Figure 1:**
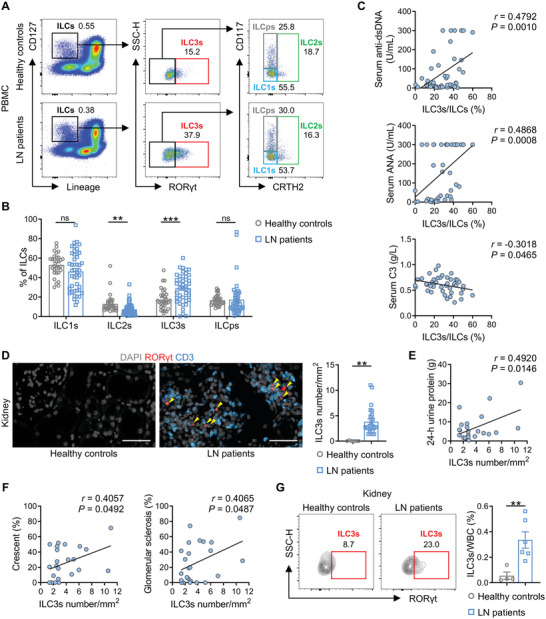
ILC3s are increased in PBMC and kidneys of LN patients. A) Representative plots of ILC subsets in human blood. B) Percentages of ILC subsets among total ILCs were compared between healthy controls (*n* = 30) and LN patients (*n* = 44). C) Correlation analysis of circulating ILC3s frequency with serum anti‐dsDNA antibodies, ANA and C3 in LN patients (*n* = 44). ANA values ≥ 300 U mL^−1^ were uniformly recorded as 300 U mL^−1^ in the test reports. D) Representative immunofluorescence staining of ILC3s in paraffin‐embedded specimens of kidneys between healthy controls and LN patients. Sections were stained for DAPI (gray), RORγt (red), and CD3 (blue). Arrows indicate ILC3s (CD3^−^RORγt^+^). Scale bars, 50 µm. The numbers of renal ILC3s per mm^2^ kidney tissue area between healthy controls (*n* = 6) and LN patients (*n* = 24) were compared. E,F) Correlation between the number of ILC3s per mm^2^ kidney tissue and 24‐h urine protein, and the percentage of glomeruli with crescents and glomerular sclerosis (*n* = 24). G) Representative flow cytometry plots and statistical data showing the percentage of ILC3s (CD45^+^Lineage^−^CD127^+^RORγt^+^) within CD45^+^ white blood cells (WBC) in renal biopsies between healthy controls (*n* = 4) and LN patients (*n* = 6). Data are shown as the mean ± SEM. Student's *t* test (B,D,G) and Pearson correlation test (C,E,F) were performed. ***P < 0.001; **P < 0.01; ns, not significant.

To explore the characteristics of ILCs in peripheral blood mononuclear cells (PBMC) of LN patients, a total of 44 LN patients and 30 healthy controls (HCs) were included in the analysis (Table S1, Supporting Information). Based on a panel of well‐recognized markers (**Figure** **1**A; Figure S1, Supporting Information),^[^ ^12^ ^]^ total ILCs from human PBMC were identified as CD45^+^lineage^−^CD127^+^ lymphocytes and further subdivided accordingly: RORγt^+^ ILC3s, RORγt^−^CRTH2^+^ ILC2s, RORγt^−^CRTH2^−^CD117^−^ ILC1s, and RORγt^−^CRTH2^−^CD117^+^ ILC progenitors (ILCps). Flow cytometry analysis revealed no difference between HCs and LN patients in either the proportion of circulating ILCs among lymphocytes (Figure S2, Supporting Information) or the proportions of ILC1s and ILCps among total ILCs (Figure 1B). In contrast, ILC2s in LN blood exhibited a relative reduction, while ILC3s was the only subpopulation that increased significantly (from ≈17% to ≈29%) (Figure 1B). Of particular importance, the percentage of circulating ILC3s in ILCs was positively correlated with serum anti‐double‐stranded DNA (anti‐dsDNA) antibodies and antinuclear antibodies (ANA), and negatively correlated with serum C3 level (Figure 1C). These results suggest that ILC3s may serve as a biomarker of disease activity. We also examined the expression of certain pro‐inflammatory subset markers, such as natural cytotoxicity receptor (NCR) marker NKp44,^[^ ^13^ ^]^ on circulating ILC3s. Despite a very limited proportion of NKp44^+^ ILC3s, their level was significantly elevated (from ≈0.4% to ≈0.9%) in LN patients compared with HCs (Figure S3, Supporting Information and Table S2, Supporting Information), indicating a rising concentration of inflammatory ILC3s in LN circulation.

Using widely employed markers to identify ILC3 cells in situ,^[^ ^14^ ^]^ we analyzed ILC3s in kidney sections. Consistent with the findings in circulation, immunofluorescence results showed a remarked accumulation of CD3^−^RORγt^+^ ILC3s in the kidneys of LN patients compared to HCs (Figure 1D and Table S3, Supporting Information). Moreover, the density of ILC3s in kidney tissues showed a positive association with 24‐h urine protein in LN patients (Figure 1E), and likewise, with the histological manifestations of severe glomerular damage, represented by the percentages of crescents and glomerular sclerosis (Figure 1F). To further elucidate ILC3s’ changes in the kidney, we detected ILC3s by flow cytometry in lupus kidney biopsies from a small group of patients, which showed an impressive increase in the proportion of ILC3s in renal leukocytes (Figure 1G and Table S4, Supporting Information). Taken together, these data indicate that ILC3s may be a potential participant in kidney damage during LN.

### 2.2 Systemically Elevated ILC3s Correlate with Serum Autoantibody Levels and Nephritis Severity in MRL/*lpr* Mice

**Figure 2:**
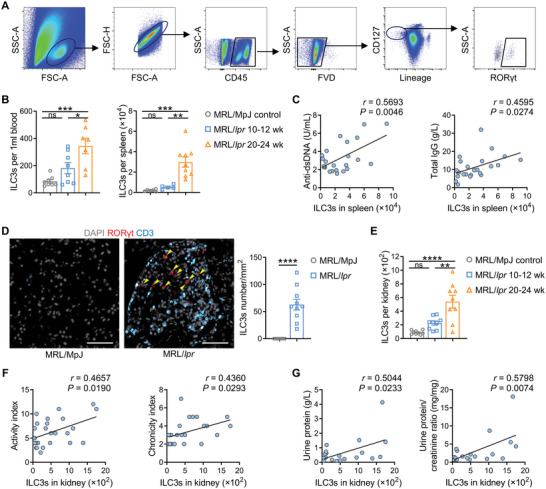
Systemically elevated ILC3s correlate with serum autoantibody level and nephritis severity in MRL/*lpr* mice. A) Gating strategy used to identify ILC3s in mice. Lineage: CD3, Ly‐6G/Ly‐6C, CD11b, B220, TER‐119. B) Quantitative analysis of ILC3s numbers in PBMC and spleens of MRL/MpJ and MRL/*lpr* mice at the indicated ages by flow cytometry (*n* = 5–9 per group). C) Correlations between the number of splenic ILC3s with levels of serum anti‐dsDNA antibody and total IgG detected by ELISA (*n* = 23). D) Representative confocal microscopy images of kidney sections from MRL/MpJ and MRL/*lpr* mice stained for DAPI (gray), RORγt (red), and CD3 (blue). Arrows indicate ILC3s (CD3^−^RORγt^+^). Scale bars, 50 µm. The numbers of renal ILC3s per mm^2^ kidney tissue area were compared (*n* = 7–10 per group). E) Absolute numbers of renal ILC3s in MRL/MpJ and MRL/*lpr* mice at the indicated ages were measured by flow cytometry (*n* = 9 per group). F) Correlations between the absolute number of renal ILC3s with LN activity index and chronicity index (*n* = 25). G) Correlations between the absolute number of renal ILC3s with urine protein and urine protein/creatinine ratio (*n* = 20). Data are shown as mean ± SEM. One‐way ANOVA test (B,E), Student's *t* test (D) and Pearson correlation test (C,F,G) were performed. ****P < 0.0001; ***P < 0.001; **P < 0.01; *P < 0.05; ns, not significant.

To further elucidate the dynamics and role of ILC3s in the process of LN, we utilized MRL/*lpr* mice, a classical murine model of spontaneous SLE/LN. ILC3s in MRL/MpJ healthy control mice, early‐stage (10–12 weeks old) and late‐stage disease (20–24 weeks old) MRL/*lpr* mice were respectively detected by flow cytometry, gated as CD45^+^lineage^−^CD127^+^RORγt^+^ lymphocytes (**Figure** **2**A). As LN developed, the numbers of ILC3s in PBMC and spleen dramatically increased (Figure 2B), taking the observation in spleen as an example, the number of ILC3s was positively correlated with serum anti‐dsDNA antibodies and total IgG concentrations (Figure 2C).

Concordantly with kidney tissues from LN patients, immunofluorescence staining revealed a marked increase in the number of CD3^−^RORγt^+^ ILC3s in lupus mice (Figure 2D). Flow cytometric analysis further confirmed a marked rise in renal ILC3s with the development of LN, especially a 6.1‐fold increase at the late‐stage of disease progression compared to controls (Figure 2E). Moreover, the trend in the amount of ILC3s present in the kidney was in great agreement with that observed in the circulation (Figure S4, Supporting Information) and, crucially, positively correlated with renal pathological indices (activity index and chronicity index) and the severity of renal dysfunction (urine protein level and urine protein/creatinine ratio) (Figure 2F,G). These results together demonstrated that systemic ILC3s were significantly elevated with LN progression, likely involved in the autoimmune response and severe nephritis phenotype. In addition to the spontaneous LN model, accumulation of renal ILC3s was also verified in nephrotoxic serum nephritis (NTN) (Figure S5, Supporting Information), an induced LN model characterized by crescent formation, leukocyte infiltration and renal functional impairment.^[^ ^15^ ^]^

### 2.3 Intestinal ILC3s are an Important Source of ILC3s Recruited to the Lupus Kidney

**Figure 3:**
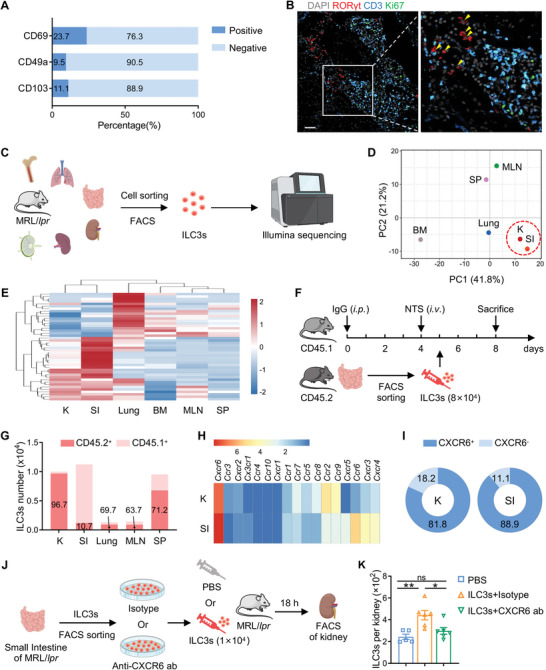
Intestinal ILC3s are an important source of ILC3s recruited to the lupus kidney. A) Expressions of resident cell markers (CD69, CD49a, CD103) on renal ILC3s of MRL/*lpr* mice were measured by flow cytometry (*n* = 6). B) Representative confocal microscopy images of kidney in MRL/*lpr* mice, with DAPI (gray), RORγt (red), CD3 (blue), and Ki67 (green). Arrows indicate ILC3s (CD3^−^RORγt^+^). Scale bar, 50 µm. C) Schematic shows the experimental design of Smart‐seq2 sequencing for ILC3s. D,E) Principal component analysis and heatmap of the transcriptome profiling of ILC3s sorted from different organs based on the gene ontology (GO) term “immune system process” by Smart‐seq2 (*n* = 3 mice per group). F) CD45.1 mice were induced NTN model and adoptive transferred with sorted intestinal ILC3s from the intestines of CD45.2 mice. G) The number of CD45.1^+^ ILC3s and CD45.2^+^ ILC3s in different organs was analyzed by flow cytometry 3 d after transfer (*n* = 3). H) Expression levels (FPKM) of chemokine receptor genes expressed by renal and intestinal ILC3s based on Smart‐seq2 (*n* = 3 per group). I) Expressions of CXCR6 on renal and intestinal ILC3s of MRL/*lpr* mice were measured by flow cytometry (*n* = 4). J) MRL/*lpr* mice were adoptive transferred with PBS or sorted intestinal ILC3s incubated with CXCR6 antibody or isotype. K) The number of renal ILC3s was analyzed by flow cytometry 18 h after transfer (*n* = 5–6 per group). Data are shown as mean ± SEM. One‐way ANOVA test (K) was conducted. **P < 0.01; *P < 0.05; ns, not significant.

ILCs are generally considered to be tissue‐resident cells^[^ ^16^ ^]^ and possess a potent proliferative capacity under inflammatory conditions,^[^ ^17^ ^]^ although several studies have also reported the migratory properties of ILCs.^[^ ^18^ ^]^ To ascertain whether the increased ILC3s in LN kidneys are resident or recruited, we first examined tissue‐resident cell markers (CD69, CD49a and CD103)^[^ ^19^ ^]^ and proliferation marker (Ki67) on ILC3s by flow cytometry or immunostaining (**Figure** **3**A,B). Surprisingly, only a handful of renal ILC3s expressed those markers (Figure 3A,B), suggesting that they do not primarily originate from proliferative kidney‐resident cells, but may instead migrate from other sites during LN progression. To test this hypothesis and identify potential source of ILC3s in LN kidney, we used a series of classical surface markers (Figure S6, Supporting Information) to sort ILC3s from different tissues including kidney (K), small intestine (SI), lung, bone marrow (BM), mesenteric lymph node (MLN) and spleen (SP) (Figure 3C), and then performed Smart‐seq2 sequencing, trying to find clues of origin by comparing similarities between them. In particular, besides the kidney, which we focused on, these tissues are generally considered to be the main development or distribution sites of ILCs in the body.^[^ ^16^ ^]^ As shown by principal component analysis (PCA) and heatmap of transcriptome profiling, ILC3s in the kidney bored the closest resemblance in overall gene expression pattern to those in the small intestine (Figure 3D,E). Meanwhile, intestinal ILC3s were more enriched compared with other tissues and increased significantly with disease progression (Figure S7, Supporting Information), implying that intestinal ILC3s may be an important candidate source for renal ILC3s in LN. To define that LN intestinal ILC3s can migrate to the kidney, we applied a CD45.1/CD45.2 tracing experiment. When sort‐purified intestinal ILC3s from CD45.2^+^ mice were transferred into CD45.1^+^ NTN mice (Figure 3F), the distribution of CD45.2^+^ ILC3s in recipients indicated that intestinal‐derived ILC3s were predominantly trafficked into the kidneys (Figure 3G; Figure S8, Supporting Information), reflecting the fact that intestinal ILC3s are indeed able to migrate into the kidneys.

Given that chemokines are thought to be the primary factors in directing immune cells into the inflamed kidney,^[^ ^20^ ^]^ we next focused on the chemokine receptor profiles on intestinal and renal ILC3s to find more specific shared features and potential migration pathways. Interestingly, the results from Smart‐seq2 sequencing showed that ILC3s from the small intestine and kidney of MRL/*lpr* mice possessed parallel chemokine profiles and both highly expressed *Cxcr6*, apparently more outstanding than other receptors (Figure 3H). Further analysis by flow cytometry confirmed that CXCR6 was expressed on ≈80% and 90% of renal and intestinal ILC3s, respectively (Figure 3I), representing the majority of ILC3s in both tissues. To clarify our speculation that renal ILC3s migrate from the intestine under the regulation of CXCR6, LN mice were adoptively transferred with gut‐derived ILC3s pretreated with anti‐CXCR6 antibody or its isotype IgG, or transferred PBS alone as control (Figure 3J). As expected, elevated renal ILC3s were also detectable in recipient MRL/*lpr* mice 18 h after transfer of isotype‐treated intestinal ILC3s compared to PBS controls (Figure 3K). In contrast, blockade of CXCR6 resulted in nearly complete suppression of ILC3s’ enterorenal migration, at levels comparable to those in controls that transferred with PBS (Figure 3K). Combined with previous reports about the high expression of CXCR6 ligand CXCL16 in LN kidneys,^[^ ^21^ ^]^ our results collectively suggest that the intestine is an important source of renal ILC3s in LN, and CXCR6/CXCL16 may serve as a pivotal regulatory mechanism for ILC3s gut‐kidney translocation. Under this theory, intestinal ILC3s could represent renal ILC3s to a certain extent to further study their function in LN kidney.

### 2.4 ILC3s Exacerbate Manifestations of Autoimmunity and Development of LN

**Figure 4:**
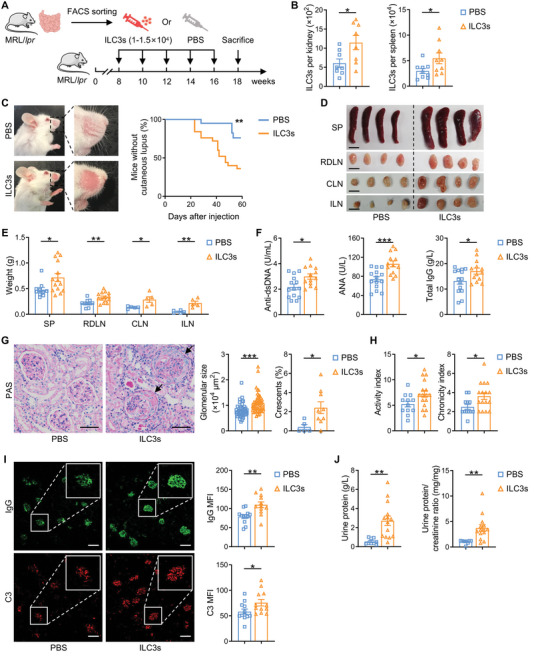
ILC3s accelerate the systemic manifestations of autoimmunity and nephritis development. A) Schematic of experimental design for adoptive transfer experiments. MRL/*lpr* mice were injected with intestinal ILC3s or PBS intravenously every two weeks from 8 to 16 wk and sacrificed at 18 wk. B) The number of ILC3s per kidney and spleen were analyzed by flow cytometry (*n* = 8–9 per group). C) Representative images of skin lesions and cumulative prevalence of MRL/*lpr* mice without cutaneous lupus (*n* = 20–25 per group). D) Representative images and E) weights of spleens (SP), renal draining lymph nodes (RDLN), cervical lymph nodes (CLN) and inguinal lymph nodes (ILN) (*n* = 5–14 per group). Scale bars, 1 cm. F) Concentrations of serum anti‐dsDNA antibody, ANA and total IgG were detected by ELISA (*n* = 13–14 per group). G) Representative images of Periodic acid‐Schiff (PAS)‐stained kidney sections. Size of glomeruli (Each plot represents a glomerulus) and proportion of crescentic glomeruli were analyzed (*n* = 5–9 per group). Arrows represent crescents. Scale bars, 50 µm. H) LN activity index and chronicity index were analyzed based on PAS‐stained kidney sections (*n* = 12–16 per group). I) Representative images of IgG (green) and C3 (red) deposition in the glomeruli. Mean fluorescence intensity (MFI) of IgG and C3 was calculated by ImageJ (*n* = 12 per group). Scale bars, 100 µm. J) Quantification of urine protein and urine protein/creatinine ratio was measured by biochemical analyzer (Roche Cobas c311) (*n* = 9–14 per group). Data are represented as mean ± SEM. Cox regression univariate analysis (C) and Student's *t* test (B and D–J) were performed. ***P < 0.001; **P < 0.01; *P < 0.05.

To further elucidate whether intestinal ILC3s play an essential role in LN progression, ILC3s from MRL/*lpr* intestines were purified and adoptively transferred into MRL/*lpr* recipient mice (**Figure** **4**A). Biweekly supplementation from 8 to 16 weeks of age resulted in a persistent elevation of both renal and splenic ILC3s (Figure 4B), representing successful up‐regulation of ILC3s in vivo. Strikingly, typical spontaneous cutaneous lesions, such as alopecia areata, were observed in mice receiving ILC3s only 3 weeks post‐transfer, significantly earlier than in control mice receiving PBS. By 8 weeks after transfer, >60% of mice receiving ILC3s developed cutaneous manifestations, compared with only ≈20% of controls (Figure 4C). Besides, up‐regulation of ILC3s also led to remarkably increased size and weight of spleens and lymph nodes (including renal draining, cervical and inguinal lymph nodes) compared with controls (Figure 4D,E), as well as elevated levels of serum anti‐dsDNA antibodies, ANA and total IgG levels in MRL/*lpr* mice (Figure 4F). These results demonstrate that ILC3s severely accelerate and exacerbate the systemic autoimmune response in lupus.

In terms of nephritis phenotype, after ILC3s infusion, histopathological analysis confirmed that MRL/*lpr* mice exhibited more aggravated renal injury, manifested as enlarged glomerulus, increased crescent proportion (Figure 4G) and higher renal activity and chronicity indices (Figure 4H). Moreover, transfer of ILC3s also significantly increased the typical immune complex deposition of IgG and complement C3 in glomeruli compared to control lupus mice receiving PBS, as immunofluorescent staining revealed evidently raised fluorescence intensities of both IgG and C3 (Figure 4I). Ultimately, significantly higher levels of urine protein and urine protein/creatinine ratio were observed in ILC3s‐transferred LN mice (Figure 4J), suggesting that ILC3s exacerbated the impairment of renal function in MRL/*lpr* mice.

### 2.5 ILC3s Promote B Cells Proliferation and Differentiation, and ELS Formation in Kidneys of MRL/*lpr* Mice

**Figure 5:**
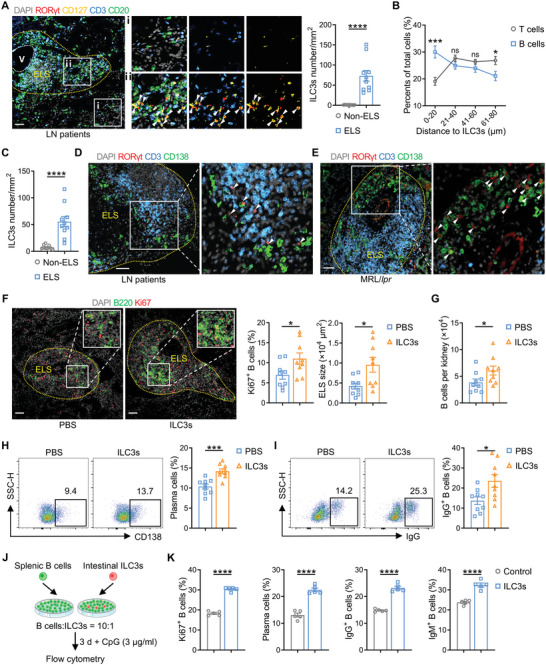
ILC3s promote B cells proliferation and differentiation, and ELS formation in kidneys of MRL/*lpr* mice. A,B) Multiplex immunofluorescence staining of LN patients’ kidney biopsies, with DAPI (gray), RORγt (red), CD127 (yellow), CD3 (blue) and CD20 (green). A) Quantification of ILC3s (CD3^−^CD127^+^RORγt^+^) in renal ELS and non‐ELS areas (*n* = 10 per group), and B) the distance of T cells and B cells to the nearest ILC3s (*n* = 35 per group) are shown. Arrows indicate ILC3s. ELS was outlined by yellow dotted lines. V, veins. Scale bar, 50 µm. C) ILC3s (CD3^−^CD127^+^RORγt^+^) in renal ELS and non‐ELS areas of MRL/*lpr* mice were counted according to immunofluorescence staining (*n* = 10 per group). D) Representative confocal microscopy images of kidney biopsies in LN patients, with DAPI (gray), RORγt (red), CD3 (blue), and CD138 (green). Arrows indicate ILC3s (CD3^−^RORγt^+^). Scale bar, 50 µm. E) Representative immunofluorescence staining of DAPI (gray), RORγt (red), CD3 (blue), and CD138 (green) in MRL/*lpr* mice kidney sections. Arrows indicate ILC3s. Scale bar, 50 µm. F–I) MRL/*lpr* mice were injected with intestinal ILC3s or PBS intravenously every two weeks from 8 to 16 wk and sacrificed at 17 wk. F) Representative immunofluorescence staining of DAPI (gray), B220 (green), and Ki67 (red) in the kidneys of MRL/*lpr* mice of the two groups. Size of ELS and percentages of proliferating B cells (Ki67^+^B220^+^) among total B cells (B220^+^) were analyzed (*n* = 9 per group). Scale bars, 50 µm. G) Numbers of B cells, H) Frequencies of plasma cells (B220^+^CD19^−^CD138^+^) and I) IgG^+^ B cells (B220^+^IgG^+^) in total B cells of kidneys were compared by flow cytometry (*n* = 9 per group). J) B cells sorted from spleen were cultured alone or co‐cultured with intestinal ILC3s for 3 d stimulated with CpG DNA (3 µg ml^−1^) to detect B cell proliferation or differentiation respectively. K) Frequencies of Ki67 ^+^ B cells, plasma cells, IgG^+^ B cells and IgM^+^ B cells were evaluated by flow cytometry (*n* = 5 per group). Data are represented as mean ± SEM. Student's *t* test was performed. ****P < 0.0001; ***P < 0.001; *P < 0.05; ns, not significant.

Using multiple immunohistochemistry to analyze the localization of human kidney ILC3s, we found that CD3^−^CD127^+^RORγt^+^ ILC3s predominantly resided within the ELS in the kidneys of LN patients, which were mainly constituted by a large number of CD3^+^ T cells and CD20^+^ B cells (**Figure** **5**A). Impressively, further analysis of the adjacency of ILC3s populations to B and T cells, respectively, in renal ELS revealed a stronger spatial relationship of ILC3s to B cells rather than T cells. Most B cells were located within a distance of 0 to 40 µm around ILC3s (of which ≈30% within 0 to 20 µm and ≈25% within 21 to 40 µm), while in the same tissue environment, more than half of T cells located >40 µm away from ILC3s (≈26% within 41 to 60 µm and ≈27% within 61–80 µm) (Figure 5B). Consistently, we could also observe the significant aggregation of ILC3s in ELS in MRL/*lpr* kidneys (Figure 5C). Likewise, renal ILC3s of both LN patients and MRL/*lpr* mice were surrounded by plenty of B cell subsets CD138^+^ plasma cells (Figure 5D,E), further implicating that ILC3s may have certain spatial effects on B cells.

To determine whether ILC3s have a putative role in promoting B cell activation in LN, we first examined the proliferation and expansion of B cells in kidneys of MRL/*lpr* mice after ILC3s up‐regulation. Following the commonly used immunostaining markers,^[^ ^22^ ^]^ we found that transferred ILC3s induced an increase in Ki67^+^ proliferative B cells and a marked enlargement of ELS size in LN kidneys (Figure 5F). And flow cytometry also detected that the absolute number of B cells in the recipients’ kidneys doubled (Figure 5G). Next, we assessed the impact of ILC3s up‐regulation on renal B cell differentiation and function. The results of flow cytometry showed that the proportions of B220^+^CD19^−^CD138^+^ plasma cells and IgG^+^ B cells in MRL/*lpr* kidneys were all significantly increased after ILC3s transfusion (Figure 5H,I; Figure S9, Supporting Information), indicating that ILC3s prime renal B cells for antibody‐producing function. Unexpectedly, however, up‐regulation of ILC3s did not cause significant alterations in other immune cells known to be involved in disease progression,^[^ ^23^ ^]^ such as Th17s, follicular helper T cells (Tfhs), regulatory T cells (Tregs), macrophages and neutrophils, within LN kidneys and multiple organs (Figure S10, Supporting Information).

To further elucidate the most direct effect of ILC3s on B cells proliferation, maturation and antibody production in the context of lupus, B220^+^ B cells sorted from the spleen were co‐cultured ex vivo with or without small intestine‐derived ILC3s at a ratio of 10:1 for 3 days (Figure 5J). This system was accompanied by the presence of CpG DNA, a classical inducer of B cell activation, which has been widely used in studies of lupus autoantibody secretion.^[^ ^24^ ^]^ Pronouncedly, higher proportions of proliferating B cells, plasma cells and IgG/IgM antibodies‐producing B cells were observed in the co‐culture model (Figure 5K; Figure S11, Supporting Information), indicating that ILC3s promote the activation process and effector functions of B cells. Combined with the positive correlation we observed between circulating ILC3s and serum autoantibody levels in humans (Figure 1C), evidences above supported the role of ILC3s in promoting B cells differentiation into plasma cells and antibodies production, as well as B cell expansion and ELS formation in the kidney, ultimately accelerated LN progression.

### 2.6 ILC3s Activate B Cells in a DLL1/Notch‐Dependent Manner, and Blockade of DLL1 Inhibits B Cell Activation and Ameliorates LN

Considering that ILC3s are potent cytokine producers, we initially speculated that ILC3s might promote B cell activation by secreting pathogenic cytokines such as IL‐17, IL‐22, TNF‐α and GM‐CSF.^[^ ^25^ ^]^ However, results from cytokine bead arrays showed that no elevated expression of a series of relevant cytokines was detected in ILC3s‐upregulated kidney tissues compared with untreated LN (Figure S12, Supporting Information). Accordingly, the role of renal ILC3s in regulating B cells here is likely not mediated by canonical cytokines. To determine how ILC3s activate B cells in LN, we further performed Smart‐seq2 sequencing to profile the gene expression of ILC3s from MRL/*lpr* kidneys at different disease stages.

**Figure 6:**
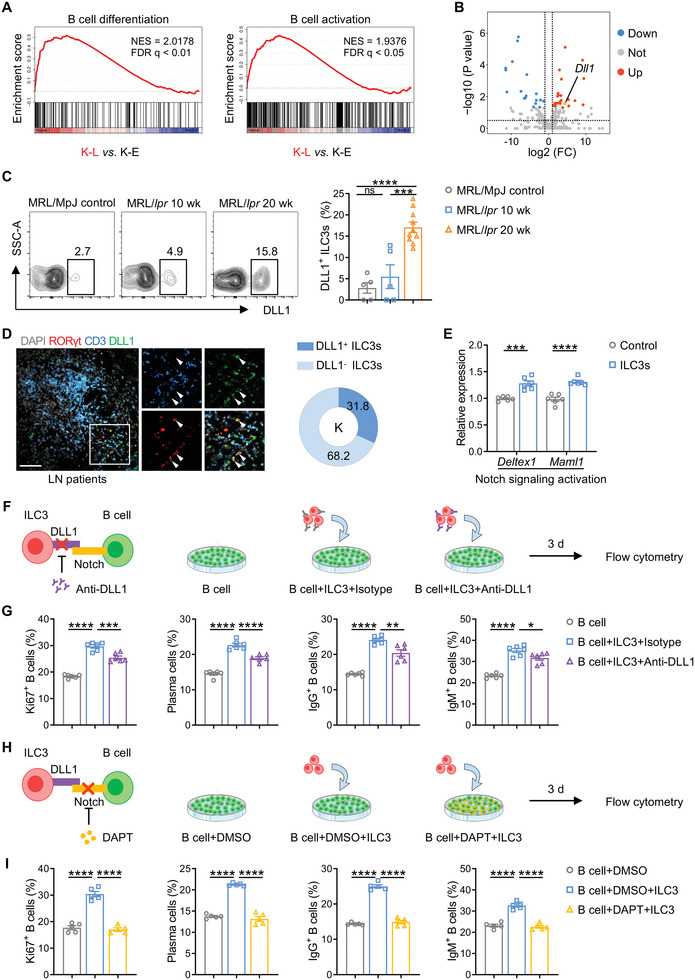
ILC3s activate B cells in a DLL1/Notch‐dependent manner. A) GSEA plots showing enrichment of gene signatures associated with “B cell differentiation” and “B cell activation” between renal ILC3 from 10‐wk‐old MRL/*lpr* mice (K‐E) and 20‐wk‐old MRL/*lpr* mice (K–L) based on Smart‐seq2 data (*n* = 3 per group). NES, normalized enrichment score. FDR, false‐discovery rate. B) Volcano plot for differentially expressed genes of renal ILC3s from 10‐wk‐old and 20‐wk‐old MRL/*lpr* mice among genes related to B cells based on Smart‐seq2 data (*n* = 3 per group). C) The proportion of renal DLL1^+^ ILC3s in total ILC3s (CD45^+^Lineage^−^CD127^+^RORγt^+^) of MRL/MpJ and MRL/*lpr* mice in the indicated ages was detected by flow cytometry (*n* = 5–10 per group). D) Expressions of DLL1 on renal ILC3s of patients with LN were measured by immunofluorescence analysis (*n* = 10), with DAPI (gray), RORγt (red), CD3 (blue) and DLL1 (green). Arrows indicate DLL1^+^ ILC3s (CD3^−^RORγt^+^DLL1^+^). Scale bars, 50 µm. E) Splenic B cells were cultured alone or co‐cultured with intestinal ILC3s for 2 d with CpG DNA (3 µg ml^−1^), then real‐time PCR analysis of *Deltex1* and *Maml1* transcript in B cells was performed (*n* = 6 per group). F,G) Splenic B cells were cultured alone or co‐cultured with intestinal ILC3s in the presence of blocking antibody against DLL1 or IgG isotype control (20 µg ml^−1^) for 3 d with CpG DNA (3 µg ml^−1^), and then frequencies of Ki67 ^+^ B cells, plasma cells, IgG^+^ B cells and IgM^+^ B cells in B cells were evaluated by flow cytometry (*n* = 6 per group). H,I) Splenic B cells were cultured alone or co‐cultured with intestinal ILC3s in the presence of 10 µM DAPT (Notch inhibitor) or DMSO vehicle control for 3 d with CpG DNA (3 µg ml^−1^), and then frequencies of Ki67^+^ B cells, plasma cells, IgG^+^ B cells and IgM^+^ B cells in B cells were evaluated by flow cytometry (*n* = 5 per group). Data are shown as mean ± SEM. Student's *t* test (E) and One‐way ANOVA test (C,G,I) were performed. ****P < 0.0001; ***P < 0.001; **P < 0.01; *P < 0.05; ns, not significant.

**Figure 7:**
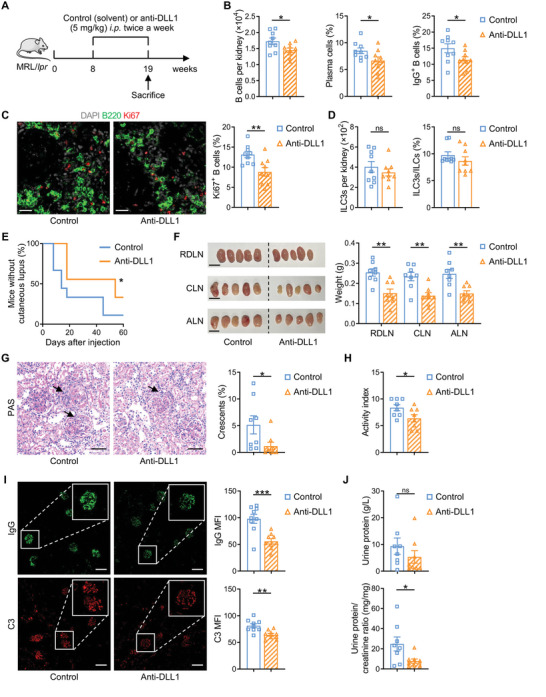
Blockade of DLL1 inhibits B cell activation and ameliorates systemic lesions and renal injury in lupus mice. A) Experiment scheme illustrating DLL1 blockade in vivo. MRL/*lpr* mice were injected with control (solvent) or anti‐DLL1 (5 mg k^−1^g) intraperitoneally twice a week from 8 to 19 wk and sacrificed at 19 wk. B) Numbers of B cells, frequencies of plasma cells and IgG^+^ B cells in total B cells in the kidneys of MRL/*lpr* mice treated with control solvent or anti‐DLL1 (*n* = 9 per group) were compared by flow cytometry. C) Representative immunofluorescence staining of DAPI (gray), B220 (green), and Ki67 (red) in the kidneys of MRL/*lpr* mice treated with control solvent or anti‐DLL1. Percentages of proliferating B cells (Ki67^+^B220^+^) among total B cells (B220^+^) were analyzed (*n* = 9 per group). Scale bars, 25 µm. D) The number and proportion of renal ILC3s in the DLL1 blockade experiment. E) Cumulative prevalence of MRL/*lpr* mice without cutaneous lupus (*n* = 9 per group). F) Representative images and weights of renal draining lymph nodes (RDLN), cervical lymph nodes (CLN) and axillary lymph nodes (ALN) (*n* = 8 per group). Scale bars, 1 cm. G) Representative images of PAS‐stained kidney sections. Proportion of crescentic glomeruli was analyzed (*n* = 8 per group). Arrows represent crescents. Scale bars, 50 µm. H) LN activity index was analyzed based on PAS‐stained kidney sections (*n* = 8 per group). I) Representative images of IgG (green) and C3 (red) deposition in the glomeruli. MFI of IgG and C3 was calculated by ImageJ (*n* = 9 per group). Scale bars, 100 µm. J) Quantification of urine protein and urine protein/creatinine ratio measured by biochemical analyzer (Roche Cobas c311) (*n* = 8 per group). Data are represented as the mean ± SEM. Student's *t* test (B–D and F–J) and Cox regression univariate analysis (E) were performed. ***P < 0.001; **P < 0.01; *P < 0.05; ns, not significant.

Using gene set enrichment analysis (GSEA), we noticed increased gene expression in renal ILC3s Gene Ontology (GO) terms “B cell differentiation” and “B cell activation” in late‐stage LN compared to early‐stage LN mice (**Figure** **6**A). Further analysis of these gene sets found that *Dll1*, a Notch ligand reported to be involved in B cell activation and differentiation,^[^ ^26^ ^]^ was significantly over‐expressed and up‐regulated in renal ILC3s of mice with advanced LN (Figure 6B). Moreover, this progressive increase in DLL1 expression on renal ILC3s was also confirmed in the observation in vivo, as shown in the dramatic increase in the proportion of DLL1^+^ ILC3s in LN mice detected by flow cytometry (Figure 6C). In humans, we determined DLL1 expression on ILC3s in LN kidney sections. Apparently, nearly one‐third of ILC3s in renal ELS were DLL1 positive (Figure 6D; Table S5, Supporting Information). Besides, the presence of DLL1^+^ ILC3s in human blood was detected as well, and their frequency was significantly increased in PBMC from LN patients compared with HCs (Figure S13, Supporting Information and Table S2, Supporting Information). Meanwhile, in the same in vitro system described above, Notch signaling in B cells was enhanced after co‐culture with intestinal ILC3s, represented by higher expression of *Deltex1* and *Maml1* (Figure 6E), two important downstream effectors in Notch signaling pathway.^[^ ^27^ ^]^ These clues suggest that DLL1‐mediated Notch signaling may be the mechanism by which ILC3 acts on B cell. To account for this, we applied DLL1 antibody to block its expression on ILC3s (Figure 6F,G; Figure S14, Supporting Information) or treated B cells with Notch inhibitor DAPT^[^ ^28^ ^]^ (Figure 6H,I; Figure S15, Supporting Information) to block DLL1‐Notch signaling transduction between the two cells, and compared the effects of these treatments on the function of ILC3s to promote B cell proliferation and antibody production. Remarkably, both blockades abrogated ILC3s‐mediated B cells differentiation, proliferation, and antibody production, highlighting the critical role of DLL1 on ILC3s to activate B cells‐Notch signaling in ILC3s’ effect. More importantly, DLL1 antibody was used to treat LN mice (**Figure** **7**A), and the in vivo results also confirmed the inhibitory effects of DLL1‐Notch signaling blockade on renal B cells, as evidenced by a significant reduction in the number of B cells, the proportion of plasma cells and IgG^+^ B cells by flow cytometry (Figure 7B), as well as renal Ki67^+^ B cells detected by immune staining (Figure 7C). Notably, we also examined the effect of DLL1 antibody treatment on the amount of ILC3s in LN kidneys and found no reduction in the number or proportion of ILC3s in the kidney, implying that the anti‐B cell effect of DLL1 blockade was not achieved by reducing ILC3s. (Figure 7D). Thus, these findings together demonstrated that ILC3s stimulated B cells in a DLL1/Notch‐dependent manner during LN.

To understand how DLL1^+^ ILC3s relate to canonical functional subgroups of ILC3s known to be IFN‐γ‐producing or lymphopoietic, termed NCR^+^ ILC3s and CCR6^+^ lymphoid tissue inducer (LTi)‐like ILC3s,^[^ ^25^ ^]^ we detected the expression of NKp46 (NCR marker) and CCR6 on ILC3s in lupus kidneys. Flow cytometry results showed that while a small number of these canonical subsets did inhabit LN kidneys, the majority were NKp46^−^CCR6^−^ double‐negative (DN) ILC3s. Of DLL1^+^ ILC3s, almost all were DN with only a very limited proportion being CCR6^+^ (Figure S16, Supporting Information). These data suggest that DLL1^+^ ILC3s may play a leading role as a distinct subset in LN.

Based on our findings, we then investigated the therapeutic potential of blocking DLL1 on systemic lesions and renal injury of lupus. Briefly, MRL/*lpr* mice were injected intraperitoneally with anti‐DLL1 antibody or vehicle twice weekly from 8 to 16 weeks of age. Reduction of CD21^hi^CD23^low^ marginal zone B (MZB) cells among splenic B220^+^ B cells (Figure S17, Supporting Information) suggested the depletion of DLL1‐dependent MZB cells, confirming the efficacy of the DLL1 antibody in vivo.^[^ ^29^ ^]^ As expected, lupus mice treated with DLL1 antibody presented a lower incidence of cutaneous lupus (Figure 7E) and reduced sizes and weights of lymph nodes (exemplified by renal draining, cervical and axillary lymph nodes (Figure 7F). In addition, blockade of DLL1 significantly attenuated the high proportion of crescentic glomeruli in advanced LN and pathological index of active kidney injury (Figure 7G,H), while ameliorating the deposition of IgG and C3 in the glomeruli (Figure 7I). Its efficacy on renal function was also documented by the decline of the urine protein to creatinine ratio (Figure 7J). All these observations demonstrate the value of targeting DLL1 in LN treatment.

## 3 Discussion

Bridging innate and adaptive immunity, ILC3s have emerged as important participants in autoimmune diseases.^[^ ^25^ ^]^ However, little is known about their role in SLE or LN. Through the observation of peripheral blood of patients, the few existing data attempted to unveil the association between ILC3s and disease. Jiang et al.^[^ ^30^ ^]^ reported that circulating ILC3s level was increased in patients with active SLE compared with inactive SLE and positively correlated with disease severity. While others have shown reduced or unchanged levels of ILC3s in PBMC from SLE patients when compared to healthy individuals.^[^ ^31^ ^]^ Uncertainty about their alteration is most likely due to the heterogeneity of the patient population and assay markers, and the contextual differences in the treatment regimens across studies. Regardless, these studies did not explore the exact role of ILC3s in the disease or observe their relevance to the kidney, which is a primary target organ in SLE. Focusing on LN, our study is the largest to date in clinical blood samples of LN patients, demonstrating the fact that circulating ILC3s were increased during kidney damage. The current study is also the first to characterize ILC3s in tissue sections and fresh biopsies of LN kidneys and to specifically link them to manifestations occurring in the kidneys of LN patients.

Recently, Hu et al.^[^ ^11^ ^]^ showed a pathogenic role of IL‐22 in LN and proposed with superficial data that ILC3s might be the main source of IL‐22 in LN kidneys. However, this study did not provide evidence of whether and how ILC3s contribute to disease progression. To gain further insight into their roles, using well‐established murine models of lupus, we reported sustained increases in ILC3s in the kidney and in multiple organs throughout the body with disease development. In vivo testing showed that up‐regulation of ILC3s could promote systemic autoimmune responses and kidney damage, further demonstrating that ILC3s amplified and accelerated LN progression.

An unexpected finding aroused our interest, as we observed that ILC3s in LN kidneys are unlikely to be tissue‐resident cells. By presenting a cross‐tissue transcriptomic comparison of ILC3s from different body sites of LN mice, we sought to identify the origin of these aggregated ILC3s in the kidney. Excitingly, among these ILC3s‐harboring tissues, a remarkable similarity between kidney‐ and gut‐derived ILC3s was discovered in this study. We identified CXCR6 as the top expressed gene among 17 chemotaxis migration‐related genes in ILC3s at these two sites. By coincidence, CXCR6's corresponding chemokine ligand, CXCL16, has been reported to be increased in the serum and urine of LN patients and in the kidneys of LN murine models including MRL/*lpr* mice,^[^ ^21^ ^]^ thus illustrating the corresponding driving force for the axial migration of ILC3s from the intestine to the kidney. All of the above links inspired us to hypothesize that intestinal ILC3s might act as a reservoir for renal ILC3s and migrate to LN kidneys via the CXCR6/CXCL16 axis.

In fact, inter‐organ migration of ILCs has been demonstrated, e.g., ILC2s residing in the gut trafficked to the lung during infection, and ILC3s were able to migrate from the intestine to the draining mesenteric lymph nodes.^[^ ^18^ ^]^ From a broader scope, it is not surprising that intestinal immunity can cause extraintestinal damage in autoimmune diseases, such as stem‐like intestinal Th17 cells migrating to the central nervous system and inducing autoimmunity.^[^ ^32^ ^]^ To test the transport of ILC3s from gut to kidney in our context, we adoptively transferred gut‐purified ILC3s into LN mice to observe renal ILC3s elevation and the reversal effect by blocking CXCR6 expression on intestinal ILC3s. Additionally, we utilized the induced LN model NTN and traced the migration of CD45.2^+^ intestinal ILC3s in CD45.1^+^ NTN mice. Our data showed that most of CD45.2^+^ intestine‐derived ILC3s were recruited to the kidneys. By applying these methods, we obtained the true occurrence and mechanistic basis of ILC3s migration. The current evidence provides another rationale for the regulation of gut‐kidney axis, extending theories built on gut microbiota‐related studies.^[^ ^33^ ^]^ Arguably, although no direct evidence exists, there are clues to the presence of a microbiota‐ILC3s‐lupus axis in the body. For instance, the gut commensal *Lactobacillus reuteri* drove autoimmunity and renal injury in Toll‐like receptor 7 (TLR7)‐dependent lupus model,^[^ ^34^ ^]^ while interestingly, this microbiota was reported in another disease to regulate the function of ILC3s in the gut.^[^ ^35^ ^]^ Consequently, it is reasonable to speculate that ILC3s might be involved in the possible causal scenarios behind microbiome interactions with the host in LN progression. Besides, elucidating the connection between the gut microbiota and the gut‐kidney ILC3s migration is a further dimension worth exploring, so as to better formulate gut‐targeted LN immune interventions. Nonetheless, we did not demonstrate whether there are other tissue sources of renal ILC3s, and it is possible that other ILC3s‐rich organs also contribute to ILC3s accumulation in the kidney.

ILCs contribute to immunity by regulating both innate and adaptive immune cells, such as T cells,^[^ ^6^ ^]^ B cells,^[^ ^36^ ^]^ macrophages^[^ ^11^ ^]^ and neutrophils,^[^ ^37^ ^]^ which have been reported to participate in the progression of LN.^[^ ^23b^ ^]^ Strikingly, of all these immune cells, only B cells showed a marked increase after ILC3s up‐regulation, as tested by repetitive animal experiments. Here it is worth highlighting their spatial distribution, with renal ILC3s mainly located in ELS structures adjacent to B cells, especially plasma cells. Given the central role of B cells and autoantibodies, as well as ELS, known as a powerhouse, in LN pathogenesis,^[^ ^38^ ^]^ we assumed that ILC3s facilitated LN progression through activating B cells and ELS formation. Indeed, effects on B cells have been discovered in certain ILC subsets. For example, ILC3s could enhance antibody production of B cells in T cell‐dependent or T cell‐independent manners,^[^ ^39^ ^]^ and ILC2s induced IgE class switching of B cells via producing type 2 cytokines.^[^ ^40^ ^]^ Whether the pathogenic role of ILC3s on B cells exists in LN remains unknown. In our study, combining in vivo observation and in vitro validation directly on B cells, we proved that ILC3s promoted B cells proliferation, maturation and antibody production, as well as subsequent ELS enlargement, which embodied the moderating effects of ILC3s to adaptive immunity and their role as the culprit in autoimmune nephritis. Besides, it was reported that splenic ILC3s promoted MZB cells to produce IgG3 antibodies, which were characteristic of T cell‐independent extrafollicular responses.^[^ ^39a^ ^]^ In lupus, plasma cells can arise through both germinal center or extrafollicular pathways.^[^ ^41^ ^]^ It would be interesting to identify which subclass of autoantibodies and pathways of B cell response were enhanced by ILC3s in LN.

To dissect the molecular mechanisms by which ILC3s induce B cell activation, we utilized Smart‐seq2 transcriptome analysis of sort‐purified ILC3s at different stages of the disease. DLL1 attracted our attention following a series of observations linking this molecular to B cell activation. DLL1 is a member of the Notch pathway with a defined function as a critical regulator in the development of MZB cells.^[^ ^42^ ^]^ In the study of G. Magri et al.,^[^ ^39a^ ^]^ they even found DLL1 as an effector molecule mediating the maturation of MZB cells triggered by CD117^+^ ILCs in the adult normal spleens. Using an in vitro co‐culture system, here we directly uncovered that ILC3s’ effects on B cell differentiation and activation relied on DLL1‐mediated Notch signaling.

Although studies have suggested a role for Notch signaling in LN progression^[^ ^43^ ^]^ and attempted to intervene at key steps in the pathway to block disease, they did not obtain the desired results.^[^ ^44^ ^]^ So far, only one study applied DLL1 intervention in LN with an endpoint to block splenic MZB cells in lupus‐prone (NZB×NZW) F1 mice,^[^ ^44^ ^]^ which unfortunately failed. And limited to the purpose of this study, the use of neutralizing antibody lasted only two weeks, without measuring the disease phenotype. In our study, we clearly showed that blockade of DLL1 suppressed B cell activity and ultimately mitigated disease in MRL/*lpr* mice. This, combined with the fact that considerable DLL1 expression on ILC3s was also detected in both blood and kidneys of LN patients, presented that DLL1 intervention may have important clinical translational value for the disease. Therefore, targeting renal ILC3s and pharmacological inhibition of DLL1 could be vigorous therapeutic strategies for LN. However, although DLL1 treatment markedly attenuated activity index in LN mice, we did not observe a significant alleviation in chronic index (data not shown). In the future, prolonging the observation time after DLL1 treatment may be more conducive to understanding its impact on the overall course of the disease. Nonetheless, it is necessary to point out the limitation of our in vivo experiment in dissecting the exact role of ILC3s‐DLL1‐B cell‐ELS regulation. Our existing data indicated that DLL1 intervention did not affect ILC3s development and proliferation, but whether DLL1‐neutralizing treatment affects the differentiation of ILC3s themselves or affects their other functions, such as intracellular signaling activation and cytokine production, remains to be answered. In addition to affecting ILC3s, the administration of DLL1‐blocking antibodies could interfere with other cells. For example, DLL1 is expressed by stromal cells.^[^ ^27^ ^]^ Therefore, further elucidation is needed in mice with DLL1 conditional knockout in ILC3s.

## 4 Conclusion

Collectively, our data demonstrated that ILC3s were markedly increased in both LN patients and mice, especially in the kidney, and the amount of ILC3s was strongly correlated with disease severity. These accumulated ILC3s in LN kidney might traffic from the gut through CXCR6/CXCL16 axis. What's more, increased ILC3s accelerated LN progression by promoting B cell proliferation, maturation and antibody production through a DLL1/Notch‐dependent mechanism, as well as augmenting renal ELS. Therefore, ILC3s may serve as a biomarker for the severity and therapeutic target for the treatment of LN.

## 5 Experimental Section

### Patients and Specimens

PBMC were isolated from peripheral blood samples of LN patients and healthy controls (Tables S1 and S2, Supporting Information). Paraffin‐embedded or fresh kidney tissues from LN patients who underwent renal biopsy at the First Affiliated Hospital of Sun Yat‐Sen University were used for immunofluorescence analysis (Table S3 and S5, Supporting Information) or flow cytometry analysis (Table S4, Supporting Information). Human para‐carcinoma kidney tissues were selected as healthy controls. LN was diagnosed according to the American College of Rheumatology revised criteria.^[^ ^45^ ^]^ The medical data were extracted from the subjects’ electronic medical records. All participants provided the informed consent forms and this study was approved by the Ethical Committee of the First Affiliated Hospital of Sun Yat‐Sen University (Approval no. 2022(644), 2016(215)).

### Mice

MRL/MpJ‐*Fas^lpr^* (MRL/*lpr*) mice were purchased from the Shanghai Slac Laboratory Animal CO. LTD. MRL/MpJ and CD45.1 mice were purchased from the Jackson Laboratory. C57BL/6 mice were obtained from Guangdong GemPharmatech Co., Ltd. Mice were housed in specific pathogen‐free conditions in the animal facility of Sun Yat‐Sen University. Animal experiments were approved by the Animal Care and Use Committee of Sun Yat‐Sen University (Approval no. SYSU‐IACUC‐2022‐000477, SYSU‐IACUC‐2022‐001094, SYSU‐IACUC‐2022‐001579).

For induction of nephrotoxic serum nephritis, 8‐week‐old female C57BL/6 mice were preimmunized intraperitoneally (*i.p*.) with 0.5 mg sheep IgG (GenXion, China) in complete Freund's adjuvant (F5881, Sigma‐Aldrich), followed by intravenous (*i.v*.) injection of 100 µl sheep nephrotoxic serum (NTS) (PTX‐001AGBM, Probetex) 4 days later. Mice were sacrificed 8 days after NTS injection. Control groups received an equal volume of PBS.

For in vivo blockade of DLL1, MRL/*lpr* mice were intraperitoneally injected with anti‐DLL1 antibody (BE0155, Bio X Cell) at 5 mg k^−1^g or solvent at an equal volume twice a week for 10 weeks.

### Cell Isolation

For human kidney biopsies, fresh tissues were cut into small pieces and digested using Multi Tissue dissociation kit (130‐110‐201, Miltenyi Biotec) following the manufacturer's instructions. The tissues were collected in enzyme mix (1.175 ml RPMI‐1640 supplemented with 50 ul of Enzyme D, 25 ul of Enzyme R and 6.75 ul of Enzyme A) and incubated for 1 h at 37 °C. Following digestion, the solution was passed through 70 µm cell strainers, centrifuged, and suspended.

In mice, tissues were harvested following perfusion with PBS. Kidney and lung tissues were minced and incubated in digestion buffer (0.05% Dispase, 0.1% Type II Collagenase for kidney or Type I Collagenase for lung, 2% FBS, RPMI‐1640) at 37 °C with shaking at 200 rpm for 30–45 min. Following digestion, cell suspensions were filtered with 70 µm cell strainers, centrifuged, and suspended in PBS.

Small intestines were washed and the contents were rinsed with PBS. After the fat and Peyer's patches were removed, the small intestines were turned inside out and cut into 1 cm pieces. Then the intestinal tissues were incubated in extraction buffer (1 mM DTT, 5 mM EDTA, 2% FBS, RPMI‐1640) at 37 °C with shaking at 200 rpm for 15 min. The remaining tissues were then washed in PBS, cut up, and incubated in digestion buffer (0.05% Dispase, 0.1% Type II Collagenase, 2% FBS, RPMI‐1640) at 37 °C with shaking at 200 rpm for 30–45 min. Following digestion, liberated cells were filtered and suspended with PBS.

Spleens, lymph nodes and bone marrow were processed by passing them through 70 µm cell strainers, and red blood lysis was performed where necessary. PBMC from human participants or mice were isolated from whole blood by Ficoll‐Paque density gradient centrifugation (GE Healthcare).

### Flow Cytometry and Cell Sorting

Flow cytometric analysis was performed according to standard protocols. Cell surface markers were stained with specific antibodies. The fixable viability dyes eFluor506 or eFluor780 (Thermo Fisher Scientific) were used to exclude dead cells. For intracellular cytokine measurements, the cells were stimulated with Cell Stimulation Cocktail (eBioscience) for 4 h. IC Fixation Buffer and Permeabilization Buffer (eBioscience) were used for intracellular staining. Intranuclear staining was done with the FOXP3/Transcription Factor Staining Buffer Set (eBioscience). Flow cytometry analysis was performed using Attune NxT (Thermo Fisher Scientific) or CytoFLEX (Beckman). Data were analyzed using FlowJo software. For sorting, ILC3s were purified with BD influx (BD) and identified as CD45^+^Lineage^−^CD127^+^CD117^+^ (Figure S6, Supporting Information). Primary B cells were isolated from the splenocytes using CD45R (B220) MicroBeads (130‐049‐501, Miltenyi Biotec) according to the manufacturer's protocol. Purities of sorted populations were typically above 98%.

### List of Antibodies Used for Flow Cytometry

See Table S6 (Supporting Information).

### Adoptive Transfers

ILC3s (1‐1.5 × 10^4^) isolated from the small intestines of MRL/*lpr* mice were adoptively transferred into MRL/*lpr* recipient mice every two weeks for a total of five applications.

### Cell Culture

ILC3s sorted from the intestine were activated using 20 ng ml^−1^ IL‐1β (R&D Systems) and 20 ng ml^−1^ IL‐23 (R&D Systems) for 2 h. Purified B cells (10^5^/well) were plated in the flat‐bottom 96‐well plate with or without activated ILC3s at a ratio of 10:1 (B cells: ILC3s), and cultured in complete RPMI medium with 3 µg ml^−1^ CpG ODN 2395 (tlrl‐2395, InvivoGen) at 37 °C and 5% CO_2_. After 72 or 48 h, the cells were collected for assessment of B cells. For DLL1 blockade, ILC3s were incubated with 20 µg ml^−1^ anti‐DLL1 monoclonal antibody (16‐5767‐85, eBioscience) or IgG isotype control (16‐4888‐81, eBioscience). For Notch inhibition, 10 µM DAPT (HY‐13027, MCE) or DMSO (Sigma–Aldrich) were added in the medium.

### Histology, Immunofluorescence, and Multiple Immunohistochemistry

Paraformaldehyde‐fixed, paraffin‐embedded kidney sections (2 µm) were stained with PAS according to standard laboratory procedures. The severity of the renal lesions was scored using the activity index and chronicity index as described for human lupus nephritis.^[^ ^46^ ^]^ The indices, crescent formation and glomerular size were assessed by a blinded reviewer.

For immunofluorescence experiments, both frozen and paraffin sections (5 µm) were blocked with Perm/blocking buffer (0.2% Triton X‐100, and 10% donkey serum in PBS). Then, sections were stained alone or in combination with antibodies against IgG (ab150113, Abcam), C3 (ab11862, Abcam), RORγt (14‐6988‐82, eBioscience), CD3 (ab5690, Abcam), B220 (14‐0452‐82, eBioscience), CD138 (IR642, Dako), CD138 (564511, BD), Ki67 (ab15580, Abcam) and DLL1 (ab10554, Abcam) overnight at 4 °C. The sections were washed and incubated with the secondary antibodies including Alexa Fluor 546 anti‐rat, Alexa Fluor 488 anti‐rabbit, Alexa Fluor 647 anti‐rabbit and Alexa Fluor 488 anti‐mouse (Thermo Fisher Scientific) for 1 h. Nuclei were stained with 4′‐6′‐diamidino‐2‐phenylindole (DAPI, Thermo Fisher Scientific). The labeled slides were visualized under a fluorescence microscope (LSM 880 confocal, Zeiss) or Mantra System (PerkinElmer, USA). Quantitative analysis was performed with the StrataQuest version 7.1.1.129 (TissueGnostics, Austria), and MFI was calculated with ImageJ.

For quantification and localization of ILC3s, B cells and T cells in human kidneys, multiple immunohistochemistry was performed. Paraffin‐embedded sections (5 µm) were stained with antibodies against CD127 (ab259806, Abcam), RORγt (14‐6988‐82, eBioscience), CD3 (ab5690, Abcam) and CD20 (ab9475, Abcam), followed by horseradish peroxidase‐conjugated secondary antibody incubation and tyramide signal amplification (TSA) using PANO 7‐plex IHC kit (#00041 00100, Panovue, Beijing, China). The slides were microwave heat‐treated after each TSA operation. DNA was counterstained with DAPI.

### ELISA, Biochemical Tests, and Cytokine Bead Array

Serum and urine samples from individual mice were collected at the end of the experiments. Serum anti‐dsDNA, ANA and total IgG antibodies were measured by ELISA kit (Alpha Diagnostics International) according to the manufacturer's instructions. Urine protein and urine creatine were measured with a fully automatic biochemical detector (Roche cobas c311). For cytokine bead array, renal samples were lysed in the lysis buffer (Millipore) with Protease Inhibitor Cocktail (Millipore). Lysates were diluted 1:1 with Assay Buffer and quantified using the Mouse Th17 Magnetic Bead Panel kits (MTH17MAG‐47K, Millipore) according to the manufacturer's instructions. Data were analyzed using Milliplex Analyst 5.1 software (Millipore).

### Transcriptome Sequencing

Total RNA was extracted from ILC3s with TRIzol (Invitrogen). RNA integrity was analyzed with Agilent 2100 Bioanalyzer (Agilent Technologies, USA) and checked using agarose gel electrophoresis. Oligo (dT) beads were used to enrich eukaryotic mRNA and rRNAs were removed by Ribo‐Zero^TM^ Magnetic Kit (Epicentre, USA). The mRNA was then fragmented into short fragments using fragmentation buffer, followed by cDNA synthesis with random primers. Second‐strand cDNA was synthesized with DNA polymerase I, RNase H, dNTP and buffer. Then the fragmented cDNA was purified using QiaQuick PCR extraction kit (Qiagen, The Netherlands), end repaired, poly (A) added, and ligated to Illumina sequencing adapters. The ligation products were size selected, amplified, and sequenced using Illumina HiSeq2500 by Gene Denovo Biotechnology Co. (Guangzhou, China). RNASeq raw data was processed with fastp (version 0.18.0). The rRNA‐mapped reads were removed using Bowtie2 (version 2.2.8). Clean reads were mapped to the genome sequence by HISAT (version 2.2.4). The mapped reads were assembled using StringTie (version 1.3.1). Differential analysis for RNA‐Seq was carried out using DESeq 2.

### Quantitative RT‐PCR

RNA from B cells was extracted with TRIzol (Invitrogen). Complementary DNA (cDNA) was synthesized using Hiscrip® II Q RT Supermix (Vazyme), and Quantitative reverse transcription PCR (qRT‐PCR) was performed using SYBR Green Master Mix (Roche). Relative expression of target genes was calculated by comparing them to the expression of the housekeeping gene *Actb*. The primers sequences that were used for qRT‐PCR were the following: 5′‐CGTAGCTCAGAGCAACCTCAT‐3′ and 5′‐TTCATGTCTTCGTCGGGCAC‐3′ for *Maml1*, 5′‐ATCAGTTCCGGCAAGACACAG‐3′ and 5′‐CGATGAGAGGTCGAGCCAC‐3′ for *Deltex1*, 5′‐ACCCGCGAGCACAGCTTCTTTG‐3′ and 5′‐ACATGCCGGAGCCGTTGTCGAC‐3′ for *Actb*.

### Statistical Analysis

All data were presented as mean ± SEM unless stated otherwise. The sample sizes (n), probability (*P*) value, and the specific statistical test for each experiment were indicated in the figure legends. Data were analyzed and plotted using Prism (GraphPad Software). Statistical analyses were performed using Student's *t* test, Pearson correlation test, one‐way analysis of variance (ANOVA) test or Cox regression univariate analysis where appropriate. All data were analyzed using two‐tailed tests, and *P* < 0.05 was considered statistically significant.

## Conflict of Interest

The authors declare no conflict of interest.

## Author Contributions

F.L., Z.L., and H.Z. contributed equally to this work. F.L., Z.L., H.Z., and Y.Z. designed, performed and interpreted all experiments. X.H., Z.T., C.Z., and R.L. carried out data analysis. F.L., X.H., Z.T., C.Z., J.S., X.H., R.Z., and R.W. performed all animal work. R.T. and H.P. performed analysis of sequencing data. X.Z., H.Y., Y.Q., J.Y., and Q.L. collected clinical blood samples from patients. P.C. collected blood samples from health controls. X.W.,Q.W., J.L., X.X., H.M., and W.C. collected kidney biopsies from patients. C.M. collected kidney biopsies from health controls. D.F., J.L., H.X., and Y.G. carried out pathological analysis. X.L. and J.F. provided technical assistance with the experiments. F.L., Z.L., H.Z., and Y.Z. wrote the manuscript.

## Supporting information

Supporting Information

## Data Availability

The data that support the findings of this study are available in the supplementary material of this article.;
