# Supplementary material for: Group 3 Innate Lymphoid Cells Exacerbate Lupus Nephritis by Promoting B Cell Activation in Kidney Ectopic Lymphoid Structures
Source: Adv Sci (Weinh). 2023 Nov 1;10(35):2302804. doi: 10.1002/advs.202302804 (PMC10724443; doi:10.1002/advs.202302804)
Supplement: Supplementary file 1 — Supporting Information [file ADVS-10-2302804-s001.pdf]

ADVANCED SCIENCE
Open Access

Supporting Information

for *Adv. Sci.*, DOI 10.1002/advs.202302804

Group 3 Innate Lymphoid Cells Exacerbate Lupus Nephritis by Promoting B Cell Activation in Kidney Ectopic Lymphoid Structures

*Feng Li, Zhou Liang, Haojie Zhong, Xinrong Hu, Ziwen Tang, Changjian Zhu, Jiani Shen, Xu Han, Ruoni Lin, Ruilin Zheng, Ruihan Tang, Huajing Peng, Xunhua Zheng, Chengqiang Mo, Peisong Chen, Xin Wang, Qiong Wen, Jianbo Li, Xi Xia, Hongjian Ye, Yagui Qiu, Jianwen Yu, Dongying Fu, Jiaqi Liu, Rong Wang, Huixin Xie, Yun Guo, Xiaoyan Li, Jinjin Fan, Qinghua Liu, Haiping Mao, Wei Chen and Yi Zhou**

Supporting Information

Group 3 Innate Lymphoid Cells Exacerbate Lupus Nephritis by Promoting B Cell Activation in Kidney Ectopic Lymphoid Structures

*Feng Li, Zhou Liang, Haojie Zhong, Xinrong Hu, Ziwen Tang, Changjian Zhu, Jiani Shen, Xu Han, Ruoni Lin, Ruilin Zheng, Ruihan Tang, Huajing Peng, Xunhua Zheng, Chengqiang Mo, Peisong Chen, Xin Wang, Qiong Wen, Jianbo Li, Xi Xia, Hongjian Ye, Yagui Qiu, Jianwen Yu, Dongying Fu, Jiaqi Liu, Rong Wang, Huixin Xie, Yun Guo, Xiaoyan Li, Jinjin Fan, Qinghua Liu, Haiping Mao, Wei Chen, Yi Zhou**

The Supplementary Materials include:

Figure S1. Gating strategy used to identify ILCs in humans.
Figure S2. Total ILCs are not changed in PBMC of patients with LN.
Figure S3. The expression of NKp44 on ILC3s in human blood.
Figure S4. The correlation between circulating ILC3s and renal ILC3s of MRL/*lpr* mice.
Figure S5. Renal ILC3s are expanded in nephrotoxic serum nephritis mice.
Figure S6. Flow cytometry gating strategy used to sort ILC3s and the expression level of RORγt is analyzed.
Figure S7. Numbers of ILC3s in different tissues of MRL/MpJ and MRL/*lpr* mice.
Figure S8. Gating strategy of $CD45.1^+$ ILC3s and $CD45.2^+$ ILC3s in NTN mice.
Figure S9. Gating strategy of B cells in vivo.
Figure S10. Adoptive transfer of ILC3s does not affect Th17s, Tfhs, Tregs, macrophages and neutrophils in MRL/*lpr* mice.
Figure S11. Representative flow cytometric images from co-culture experiments.
Figure S12. ILC3s-related cytokines in the kidneys are not altered after up-regulation of ILC3s in MRL/*lpr* mice.
Figure S13. The expression of DLL1 on ILC3s in human blood.
Figure S14. Representative flow cytometric images from in vitro DLL1 blockade experiments.
Figure S15. Representative flow cytometric images from in vitro Notch inhibition experiments.
Figure S16. ILC3 subsets in the kidneys of LN mice.
Figure S17. Blocking effect of DLL1-neutralizing antibody in lupus mice.
Table S1. Clinical characteristics of patients tested for circulating ILC subsets.

Table S2. Clinical characteristics of patients tested for DLL1 and NKp44 expression on blood ILC3s.

Table S3. Clinical characteristics of patients detected for renal ILC3s by immunofluorescence.

Table S4. Clinical characteristics of patients detected for renal ILC3s by flow cytometry.

Table S5. Clinical characteristics of patients detected for DLL1 expression on renal ILC3s.

Table S6. Antibodies used for flow cytometry.

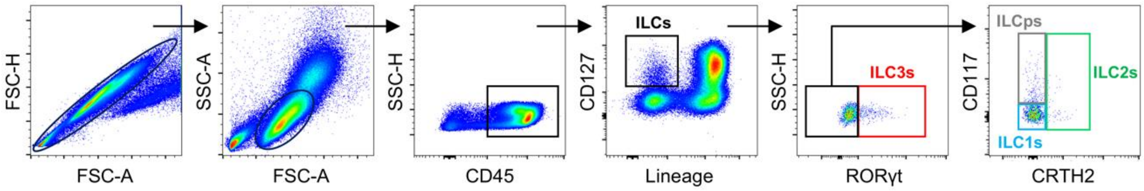

Figure S1. Gating strategy used to identify ILCs in humans. ILCs were gated as CD45^{+}Lineage^{-}CD127^{+} lymphocytes (Lineage: CD3, CD11c, CD14, CD19) in human PBMC, and further divided into individual subsets: RORγt^{+} ILC3s, RORγt^{-}CRTH2^{+} ILC2s, RORγt^{-}CRTH2^{-}CD117^{-} ILC1s, and RORγt^{-}CRTH2^{-}CD117^{+} ILC progenitors (ILCps).

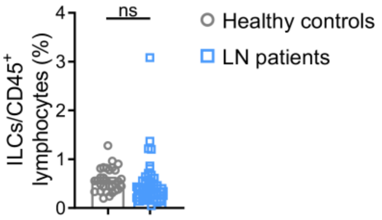

Figure S2. Total ILCs are not changed in PBMC of patients with LN. Percentages of total ILCs among CD45^{+} lymphocytes between healthy controls (n = 30) and lupus nephritis (LN) patients (n = 44) were compared. Data are shown as mean ± SEM. Student's t test was performed. ns, not significant.

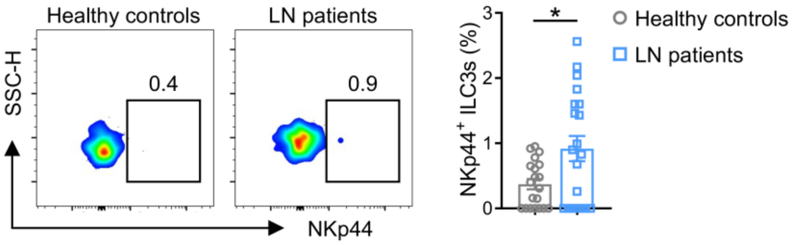

Figure S3. The expression of NKp44 on ILC3s in human blood. Flow cytometric analysis of NKp44 expression on ILC3s (CD45^{+}Lineage^{-}CD127^{+}RORγt^{+}) from PBMC of healthy controls and LN patients (n = 20 per group). Data are shown as mean ± SEM. Student's t test was performed. *P < 0.05.

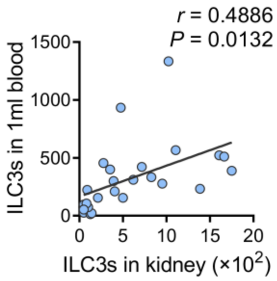

Figure S4. The correlation between circulating ILC3s and renal ILC3s of MRL/*lpr* mice. Pearson correlation between the number of ILC3s in PBMCs with that of ILC3s in kidneys of MRL/*lpr* mice was detected by flow cytometry (n = 25). Pearson correlation test was conducted.

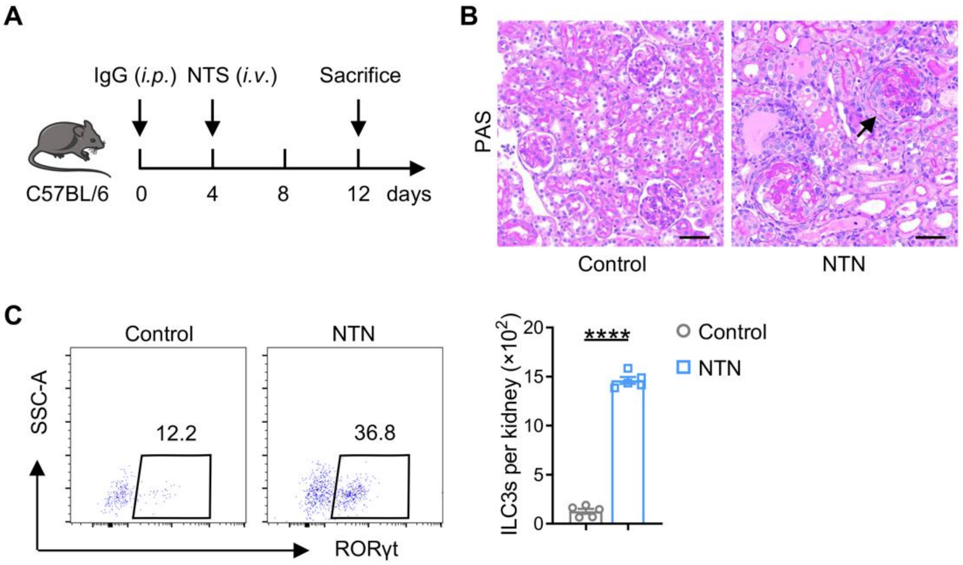

Figure S5. Renal ILC3s are expanded in nephrotoxic serum nephritis mice. A) Schematic of experimental design for nephrotoxic serum-induced LN model (nephrotoxic serum nephritis, NTN). C57BL/6 female mice were preimmunized intraperitoneally (*i.p.*) with 0.5 mg sheep IgG in complete Freund's adjuvant, followed by intravenous (*i.v.*) injection of 100 µl sheep nephrotoxic serum (NTS) 4 d later. Mice were sacrificed 8 d after NTS injection. B) Representative PAS staining of kidney sections from control and NTN mice. Arrows represent crescents. Scale bars, 50 µm. C) Representative flow cytometry plots and statistical data showing renal ILC3s ($CD45^+Lineage^-CD127^+$ $ROR\gamma t^+$) between control and NTN mice (n = 5 per group). Data are shown as mean ± SEM. Student's t test was performed. $^{****}P < 0.0001$.

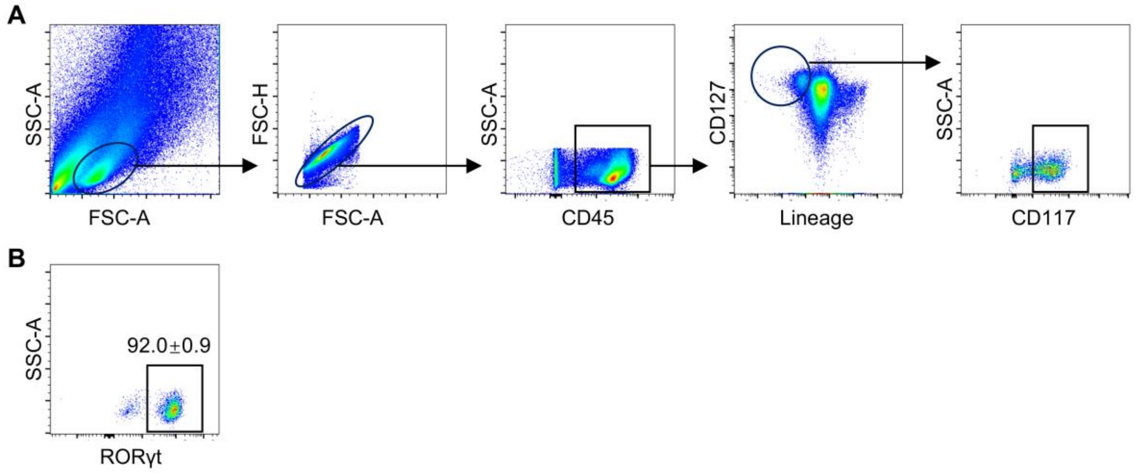

Figure S6. Flow cytometry gating strategy used to sort ILC3s and the expression level of RORγt is analyzed. A) Representative gating strategy used to sort ILC3s in mice. B) Expressions of RORγt on sorted ILC3s ($CD45^{+}Lineage^{-}CD127^{+}$ $CD117^{+}$ lymphocytes) were measured by flow cytometry (n = 4). Data are shown as mean ± SD.

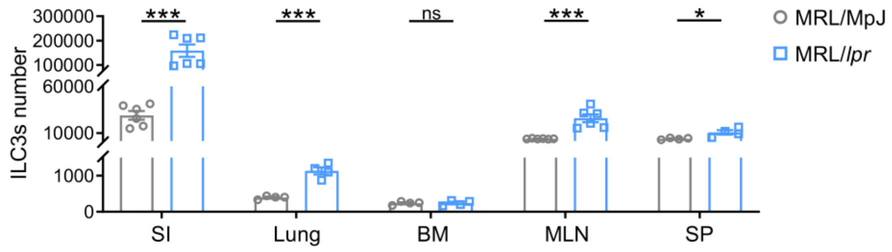

Figure S7. Numbers of ILC3s in different tissues of MRL/MpJ and MRL/*lpr* mice. Absolute numbers of ILC3s in small intestine, lung, femur bone marrow, mesenteric lymph nodes (MLN) and spleen in MRL/MpJ and MRL/*lpr* mice (n = 4-6 per group) were compared by flow cytometry. Data are shown as mean ± SEM. Student's t test was performed. ***P < 0.001; *P < 0.05; ns, not significant.

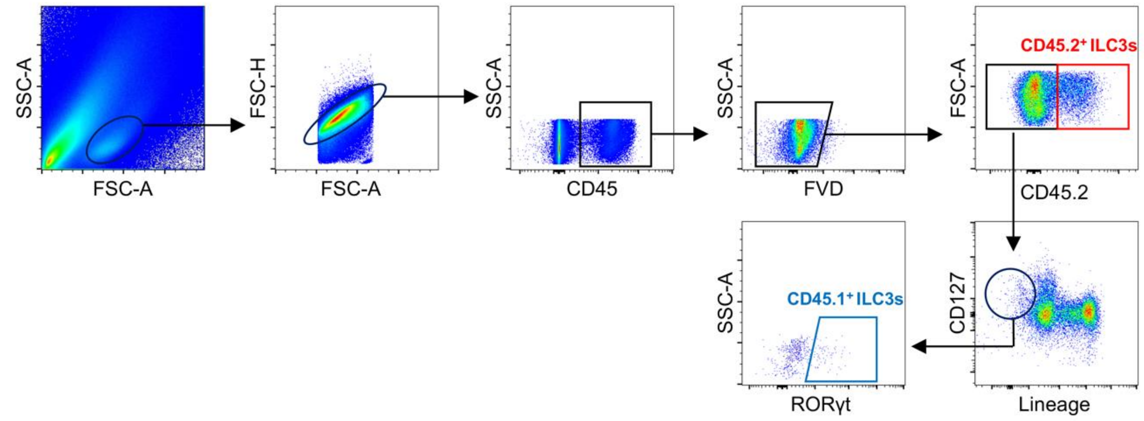

Figure S8. Gating strategy of CD45.1$^+$ ILC3s and CD45.2$^+$ ILC3s in NTN mice.

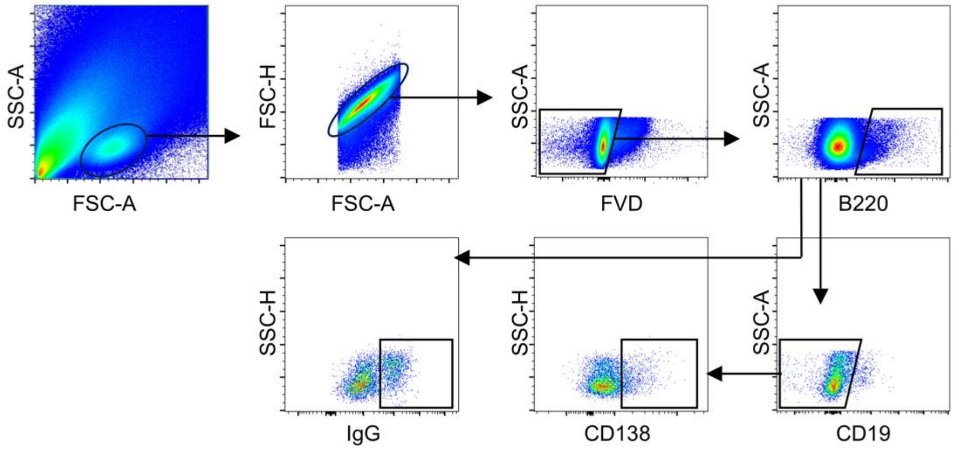

Figure S9. Gating strategy of B cells in vivo. Representative gating strategy used to identify IgG$^+$ B cells and plasma cells in MRL/*lpr* mice.

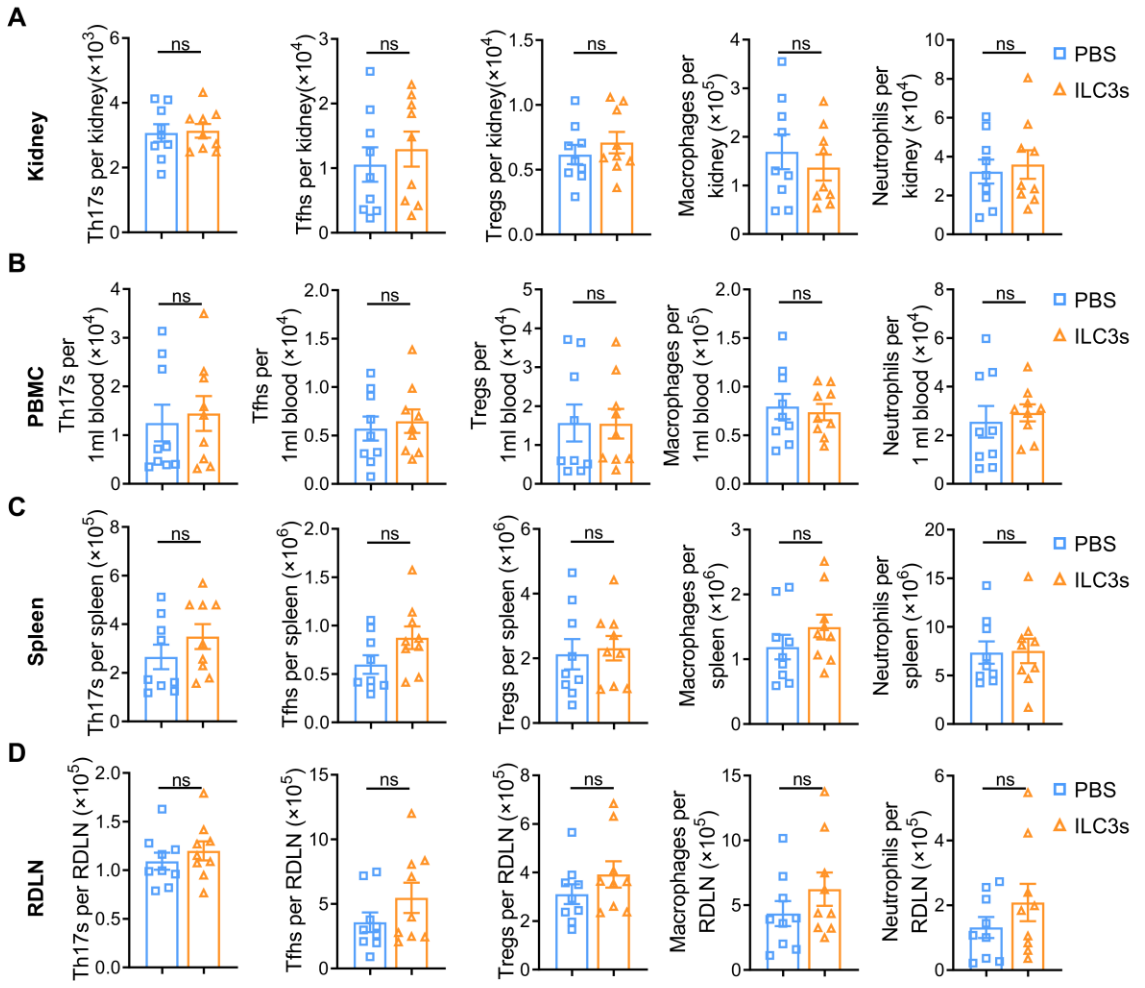

Figure S10. Adoptive transfer of ILC3s does not affect Th17s, Tfhs, Tregs, macrophages and neutrophils in MRL/*lpr* mice. MRL/*lpr* mice were transferred with intestinal ILC3s or PBS intravenously every two weeks from 8 wk to 16 wk and sacrificed at 17 wk. Absolute numbers of renal Th17s (live $CD4^+ROR\gamma t^+$), Tfhs (live $CD4^+CXCR5^+PD\text{-}1^+$), Tregs (live $CD4^+Foxp3^+$), macrophages (live $CD45^+Ly6G^-CD11b^+F4/80^+$) and neutrophils (live $CD45^+CD11b^+Ly6G^+$) in A) kidneys, B) PBMCs, C) spleens and D) renal draining lymph nodes (RDLN) were compared by flow cytometry (n = 9 per group). Data are shown as mean ± SEM. Student's *t* test was performed. ns, not significant.

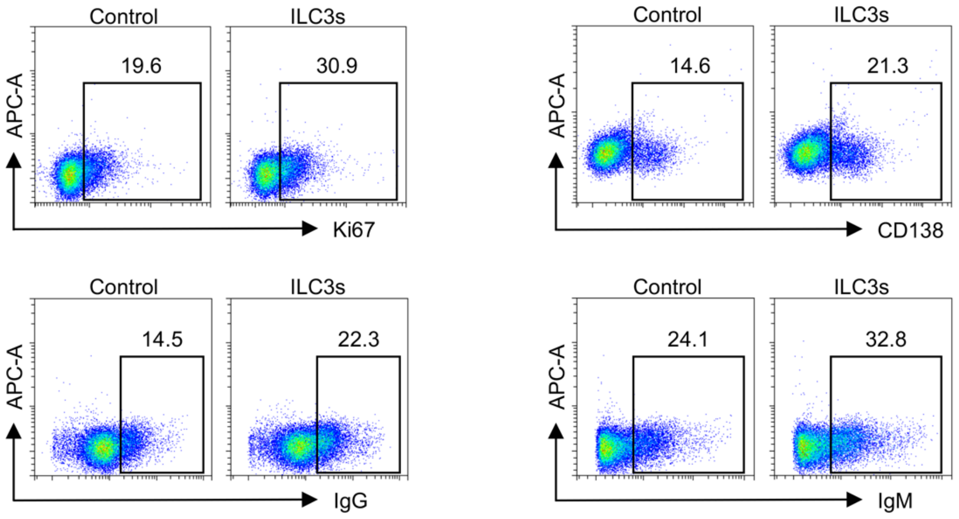

Figure S11. Representative flow cytometric images from co-culture experiments. Representative flow cytometric images showing Ki67$^+$ B cells, plasma cells, IgG$^+$ B cells and IgM$^+$ B cells in B220$^+$ B cells in the ILC3s and B cells co-culture system.

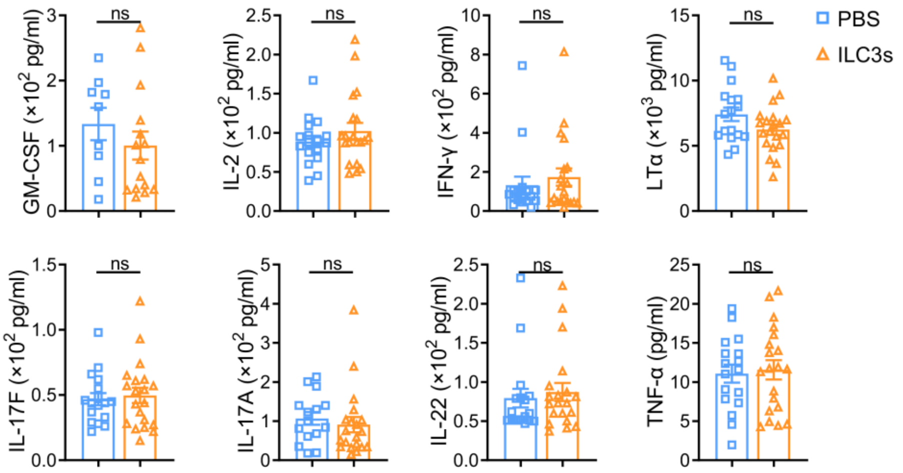

Figure S12. ILC3s-related cytokines in the kidneys are not altered after up-regulation of ILC3s in MRL/*lpr* mice. Quantitative analysis of ILC3s-related cytokines concentrations by cytokine bead array in the kidney of ILC3s-treated and PBS-treated MRL/*lpr* mice (n = 9-20 mice per group). Data are shown as mean ± SEM. Student's t test was performed. ns, not significant; LTα, lymphotoxin α.

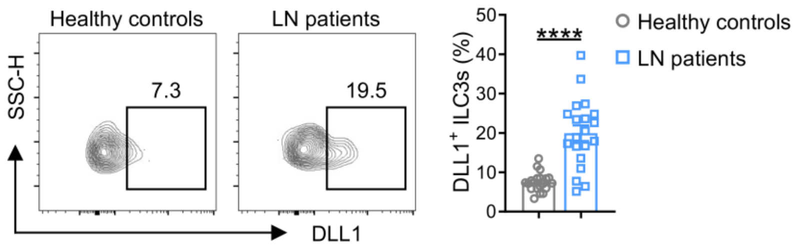

Figure S13. The expression of DLL1 on ILC3s in human blood. Flow cytometric analysis of DLL1 expression on ILC3s (CD45^{+}Lineage^{-}CD127^{+}RORγt^{+}) from PBMC of healthy controls and LN patients (n = 20 per group). Data are shown as mean ± SEM. Student's *t* test was performed. ****$P < 0.0001$.

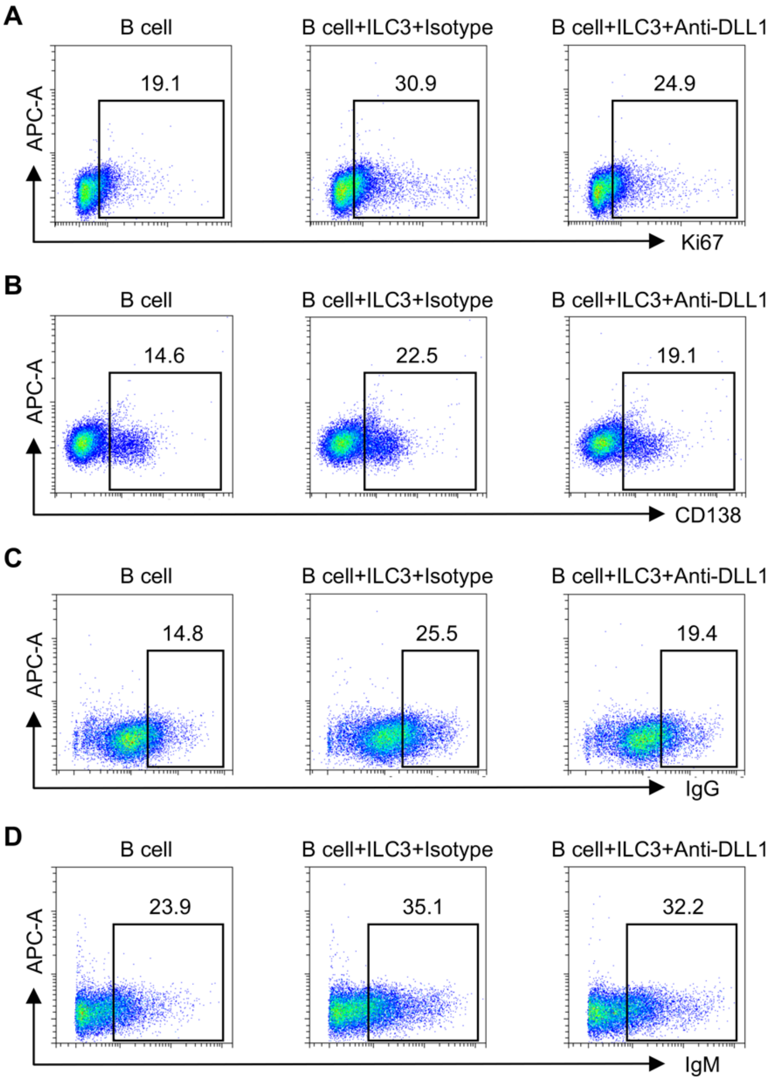

Figure S14. Representative flow cytometric images from in vitro DLL1 blockade experiments. Representative flow cytometric images showing the percentages of A) Ki67$^+$ B cells, B) plasma cells, C) IgG$^+$ B cells and D) IgM$^+$ B cells in B220$^+$ B cells of in vitro DLL1 blocking experiments.

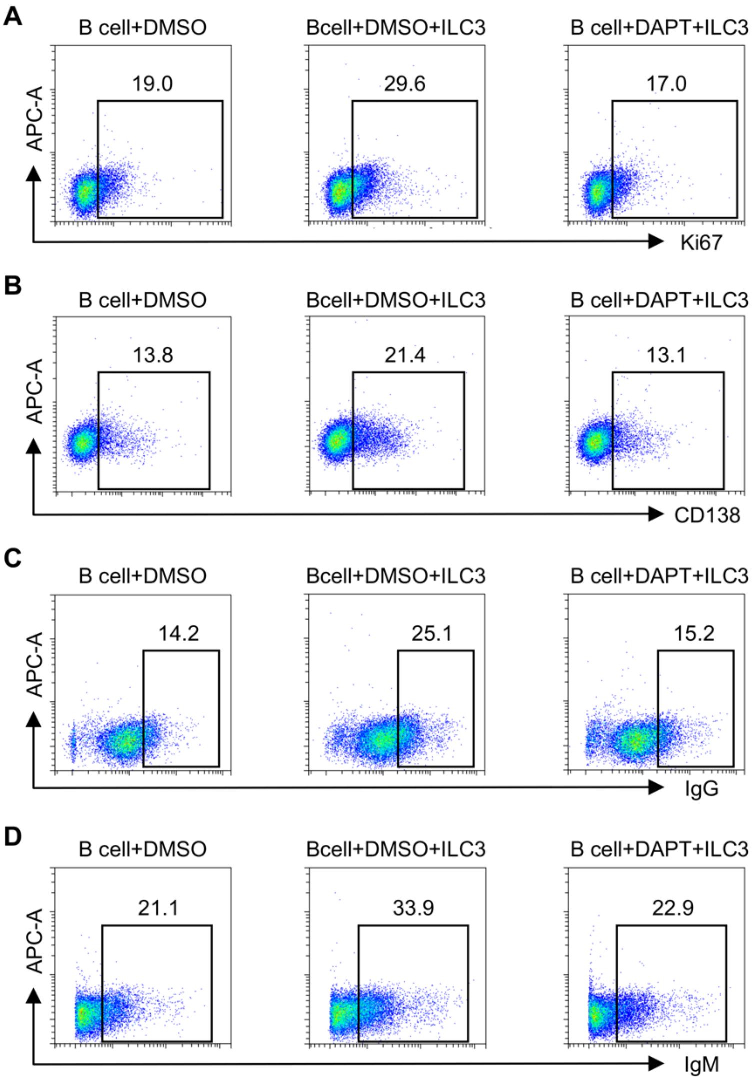

Figure S15. Representative flow cytometric images from in vitro Notch inhibition experiments. Representative flow cytometric images showing the percentages of A) Ki67^{+} B cells, B) plasma cells, C) IgG^{+} B cells and D) IgM^{+} B cells in B220^{+} B cells from the experiment of Notch inhibition in vitro.

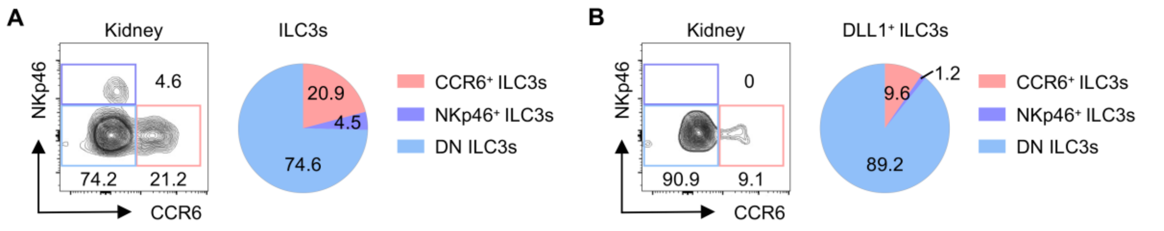

Figure S16. ILC3 subsets in the kidneys of LN mice. Representative plots and pie charts showing CCR6 and NKp46 expression on ILC3s ($CD45^{+}Lineage^{-}CD127^{+}ROR\gamma t^{+}$) (A) and $DLL1^{+}$ ILC3s ($CD45^{+}Lineage^{-}CD127^{+}ROR\gamma t^{+}DLL1^{+}$) (B) in the kidneys of *MRL/lpr* mice (n = 5). DN ILC3s, $NKp46^{-}CCR6^{-}$ double-negative ILC3s.

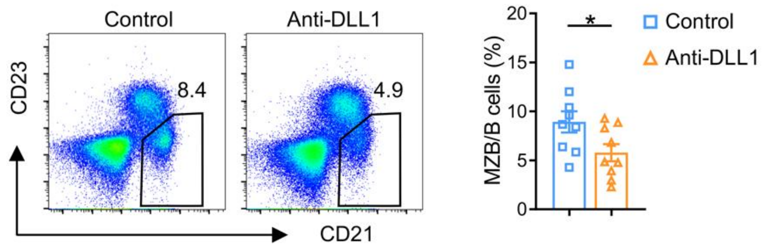

Figure S17. Blocking effect of DLL1-neutralizing antibody in lupus mice. Representative flow cytometry plots and statistical data identifying the effectiveness of DLL1-blocking antibody treatment by depletion of $CD21^{hi}CD23^{low}$ marginal zone B (MZB) cells among mature $B220^{+}$ splenic cells ($n = 9$ per group). Data are shown as mean ± SEM. Student's *t* test was performed. $^{*}P < 0.05$.

Table S1. Clinical characteristics of patients tested for circulating ILC subsets.

	Healthy controls (n = 30)	LN patients (n = 44)
Age, years	36.1 ± 8.3	33.3 ± 11.8
Female	18 (60.0%)	31 (70.5%)
Duration of SLE, years	-	5.3 ± 7.1
SLEDAI	-	12.9 ± 5.7
dsDNA, U/mL	-	79.4 ± 103.1
ANA, U/mL	-	156.8 ± 134.4
C3, g/L	-	0.6 ± 0.2
C4, g/L	-	0.2 ± 0.1
24-h urine protein, g		3.5 ± 4.8
eGFR, ml/min/1.73m^2	-	68.9 ± 44.8
Glucocorticoids	-	44 (100.0%)
Immunosuppressants	-	38 (86.4%)
ISN/RPS Class		
Unknown	-	9 (20.5%)
II		1 (2.3%)
Ⅲ	-	1 (2.3%)
Ⅳ	-	21 (47.7%)
Ⅳ+ Ⅴ	-	8 (18.2%)
Ⅴ+ Ⅲ	-	3 (6.8%)
Ⅴ	-	1 (2.3%)

Data are presented as mean ± standard deviations or n (%). SLEDAI, SLE Disease Activity Index; ANA, antinuclear antibodies; eGFR, estimated glomerular filtration rate; ISN/RPS, International Society of Nephrology/Renal Pathology Society.

Table S2. Clinical characteristics of patients tested for DLL1 and NKp44 expression on blood ILC3s.

	Healthy controls (*n* = 20)	LN patients (*n* = 20)
Age, years	32.1 ± 8.5	34.2 ± 13.2
Female	12 (60.0%)	13 (65.0%)
Duration of SLE, years	-	7.9 ± 7.8
SLEDAI	-	12.0 ± 7.5
dsDNA, U/mL	-	76.6 ± 95.2
ANA, U/mL	-	195.7 ± 140.2
C3, g/L	-	0.6 ± 0.3
C4, g/L	-	0.2 ± 0.1
24-h urine protein, g		1.2 ± 1.2
eGFR, ml/min/1.73m^2	-	90.9 ± 55.0
Glucocorticoids	-	19 (95.0%)
Immunosuppressants	-	19 (95.0%)
ISN/RPS Class		
Unknown	-	5 (25.0%)
Ⅲ	-	3 (15.0%)
Ⅳ	-	7 (35.0%)
Ⅳ+ Ⅴ	-	1 (5.0%)
Ⅴ	-	4 (20.0%)

Data are presented as mean ± standard deviations or n (%). SLEDAI, SLE Disease Activity Index; ANA, antinuclear antibodies; eGFR, estimated glomerular filtration rate; ISN/RPS, International Society of Nephrology/Renal Pathology Society.

Table S3. Clinical characteristics of patients detected for renal ILC3s by immunofluorescence.

	LN patients (n = 24)
Age, years	32.8 ± 10.8
Female	15 (62.5%)
Duration of SLE, years	3.8 ± 4.9
SLEDAI	14.6 ± 4.0
dsDNA, U/mL	189.7 ± 220.9
ANA, U/mL	160.5 ± 126.2
C3, g/L	0.5 ± 0.2
C4, g/L	0.1 ± 0.1
24h urine protein, g	6.9 ± 7.3
eGFR, ml/min/1.73m^2	42.8 ± 35.3
Glucocorticoids	19 (79.2%)
Immunosuppressants	14 (58.3%)
ISN/RPS Class	
Ⅲ	1 (4.2%)
Ⅳ	22 (91.6%)
Ⅴ	1 (4.2%)

Data are presented as mean ± standard deviations or n (%). SLEDAI, SLE Disease Activity Index; ANA, antinuclear antibodies; eGFR, estimated glomerular filtration rate; ISN/RPS, International Society of Nephrology/Renal Pathology Society.

Table S4. Clinical characteristics of patients detected for renal ILC3s by flow cytometry.

	LN patients (n = 6)
Age, years	26.7 ± 10.7
Female	4 (66.7%)
Duration of SLE, years	3.8 ± 5.6
SLEDAI	15.3 ± 3.7
dsDNA, U/mL	42.2 ± 55.8
ANA, U/mL	66.1 ± 116.4
C3, g/L	0.7 ± 0.1
C4, g/L	0.2 ± 0.1
24h urine protein, g	7.1 ± 8.0
eGFR, ml/min/1.73m^2	64.7 ± 41.2
Glucocorticoids	6 (100.0%)
Immunosuppressants	5 (83.3%)
ISN/RPS Class	
Ⅲ	1 (16.7%)
Ⅳ	1 (16.7%)
Ⅳ+ Ⅴ	2 (33.3%)
Ⅴ+ Ⅲ	2 (33.3%)

Data are presented as mean ± standard deviations or n (%). SLEDAI, SLE Disease Activity Index; ANA, antinuclear antibodies; eGFR, estimated glomerular filtration rate; ISN/RPS, International Society of Nephrology/Renal Pathology Society.

Table S5. Clinical characteristics of patients detected for DLL1 expression on renal ILC3s.

	LN patients (n = 10)
Age, years	32.0 ± 8.0
Female	7 (70.0%)
Duration of SLE, years	6.5 ± 6.9
SLEDAI	13.7 ± 4.8
dsDNA, U/mL	163.6 ± 131.3
ANA, U/mL	86.6 ± 119.1
C3, g/L	0.5 ± 0.2
C4, g/L	0.1 ± 0.1
24h urine protein, g	5.5 ± 3.7
eGFR, ml/min/1.73m^2	53.6 ± 34.3
Glucocorticoids	7 (70.0%)
Immunosuppressants	5 (50.0%)
ISN/RPS Class	
Ⅳ	10 (100.0%)

Data are presented as mean ± standard deviations or n (%). SLEDAI, SLE Disease Activity Index; ANA, antinuclear antibodies; eGFR, estimated glomerular filtration rate; ISN/RPS, International Society of Nephrology/Renal Pathology Society.

Table S6. Antibodies used for flow cytometry.

Antibodies	Source	Identifier
Anti-human CD45, PE (HI30)	Biolegend	304008
Anti-human CD3, FITC (OKT3)	Biolegend	318306
Anti-human CD11c, FITC (3.9)	Biolegend	301604
Anti-human CD14, FITC (HCD14)	Biolegend	325604
Anti-human CD19, FITC (HIB19)	Biolegend	302206
Anti-human CD127, Pacific Blue (A019D5)	Biolegend	351306
Anti-human CD56, BV605 (NCAM)	Biolegend	362538
Anti-human CRTH2, APC-Cy7 (BM16)	Biolegend	350114
Anti-human/mouse RORγt, APC (AFKJS-9)	eBioscience	17-6988-82
Anti-human CD117, PE-Cy7 (104D2)	eBioscience	25-1178-42
Anti-human CD45, APC/Fire™ 750 (HI30)	Biolegend	304061
Anti-human DLL1, PE (MHD1-314)	Biolegend	346403
Anti-human NKp44, PerCP/Cyanine5.5 (P44-8)	Biolegend	325113
Anti-mouse CD45, APC/Fire™ 750 (30-F11)	Biolegend	103154
Anti-mouse Lineage Cocktail with Isotype Ctrl	Biolegend	133302
Anti-mouse CD3ε, PE-Cy7 (145-2C11)	Biolegend	100320
Anti-mouse Ly-6G/Ly-6C, PE-Cy7 (RB6-8C5)	Biolegend	108415
Anti-mouse CD11b, PE-Cy7 (M1/70)	Biolegend	101215
Anti-mouse CD45R/B220, PE-Cy7 (RA3-6B2)	Biolegend	103221
Anti-mouse TER-119, PE-Cy7 (Ter-119.)	Biolegend	116221
Anti-mouse CD127, PE-Cy7 (A7R34)	Biolegend	135014
Anti-mouse CD127, BV421 (A7R34)	Biolegend	135024
Anti-mouse RORγt, APC (B2D)	eBioscience	17-6981-82
Anti-mouse RORγt, BV421 (Q31-378)	BD	562894
Anti-mouse CD117, PE (2B8)	Biolegend	105808
Anti-mouse CD69, Pacific Blue (H1.2F3)	Biolegend	104524
Anti-mouse CD49a, PE (HMα1)	Biolegend	142604
Anti-mouse CD103, BV605 (2E7)	Biolegend	121433
Anti-mouse CD45.2, APC (104)	Biolegend	109814
Anti-mouse CXCR6, APC (SA051D1)	Biolegend	151106
Anti-mouse CD45R/B220, APC/Fire™ 750 (RA3-6B2)	Biolegend	103260

Anti-mouse CD45R/B220, BV510 (RA3-6B2)	Biolegend	103248
Anti-mouse CD19, Pacific Blue (6D5)	Biolegend	115523
Anti-mouse CD19, FITC (6D5)	Biolegend	115506
Anti-mouse CD138, APC (281-2)	Biolegend	142506
Anti-mouse CD138, PE-Cy7 (281-2)	Biolegend	142514
Anti-mouse IgG, FITC (Poly4060)	Biolegend	406001
Anti-mouse IgM, PE-Cy7 (RMM-1)	Biolegend	406514
Anti-mouse Ki67, PE (11F6)	Biolegend	151209
Anti-mouse CD4, FITC (GK1.5)	Biolegend	100406
Anti-mouse Foxp3, PE (FJK-16S)	eBioscience	12-5773-82
Anti-mouse CXCR5, PE-Cy7 (L138D7)	Biolegend	145515
Anti-mouse PD-1, APC (RMP1-30)	Biolegend	109112
Anti-mouse Ly6G, FITC (1A8)	Biolegend	127606
Anti-mouse F4/80, APC (BM8)	eBioscience	17-4801-82
Anti-mouse DLL1, PE (HMD1-3)	Biolegend	128307
Anti-mouse DLL1, APC (HMD1-3)	Biolegend	128314
Anti-mouse CCR6, PE (29-2L17)	Biolegend	129804
Anti-mouse NKp46, Alexa Fluor™ 700 (29A1.4)	eBioscience	56-3351-82
Anti-mouse CD21/CD35, APC/Fire™ 750 (7E9)	Biolegend	123433
Anti-mouse CD23, PE (B3B4)	Biolegend	101607
